# Compression of morbidity in a progeroid mouse model through the attenuation of myostatin/activin signalling

**DOI:** 10.1002/jcsm.12404

**Published:** 2019-03-27

**Authors:** Khalid Alyodawi, Wilbert P. Vermeij, Saleh Omairi, Oliver Kretz, Mark Hopkinson, Francesca Solagna, Barbara Joch, Renata M.C. Brandt, Sander Barnhoorn, Nicole van Vliet, Yanto Ridwan, Jeroen Essers, Robert Mitchell, Taryn Morash, Arja Pasternack, Olli Ritvos, Antonios Matsakas, Henry Collins‐Hooper, Tobias B. Huber, Jan H.J. Hoeijmakers, Ketan Patel

**Affiliations:** ^1^ School of Biological Sciences University of Reading Reading UK; ^2^ College of Medicine Wasit University Kut Iraq; ^3^ Department of Molecular Genetics Erasmus University Medical Center Rotterdam The Netherlands; ^4^ Princess Máxima Center Oncode Institute Utrecht The Netherlands; ^5^ Medizinische Klinik Universitätsklinikum Hamburg‐Eppendorf Hamburg Germany; ^6^ Department of Medicine IV, Faculty of Medicine University of Freiburg Freiburg Germany; ^7^ Department of Neuroanatomy, Faculty of Medicine University of Freiburg Freiburg Germany; ^8^ Royal Veterinary College London UK; ^9^ Department of Radiology and Nuclear Medicine Erasmus MC Rotterdam The Netherlands; ^10^ Department of Radiation Oncology Erasmus MC Rotterdam The Netherlands; ^11^ Department of Vascular Surgery Erasmus MC Rotterdam The Netherlands; ^12^ Department of Bacteriology and Immunology University of Helsinki Helsinki Finland; ^13^ Institute of Molecular Medicine University of Health Science Center Houston TX USA; ^14^ Molecular Physiology Laboratory Hull York Medical School Hull UK; ^15^ BIOSS Center for Biological Signalling Studies University of Freiburg Freiburg Germany; ^16^ Freiburg Institute for Advanced Studies and Center for Biological System Analysis Freiburg Germany; ^17^ CECAD Forschungszentrum Universität zu Köln Cologne Germany

**Keywords:** Compression, Morbidity, Progeroid, Ageing, Skeletal muscle, Myostatin, Liver, Kidney, Bone, Neurological

## Abstract

**Background:**

One of the principles underpinning our understanding of ageing is that DNA damage induces a stress response that shifts cellular resources from growth towards maintenance. A contrasting and seemingly irreconcilable view is that prompting growth of, for example, skeletal muscle confers systemic benefit.

**Methods:**

To investigate the robustness of these axioms, we induced muscle growth in a murine progeroid model through the use of activin receptor IIB ligand trap that dampens myostatin/activin signalling. Progeric mice were then investigated for neurological and muscle function as well as cellular profiling of the muscle, kidney, liver, and bone.

**Results:**

We show that muscle of *Ercc1* ^Δ/−^ progeroid mice undergoes severe wasting (decreases in hind limb muscle mass of 40–60% compared with normal mass), which is largely protected by attenuating myostatin/activin signalling using soluble activin receptor type IIB (sActRIIB) (increase of 30–62% compared with untreated progeric). sActRIIB‐treated progeroid mice maintained muscle activity (distance travel per hour: 5.6 m in untreated mice vs. 13.7 m in treated) and increased specific force (19.3 mN/mg in untreated vs. 24.0 mN/mg in treated). sActRIIb treatment of progeroid mice also improved satellite cell function especially their ability to proliferate on their native substrate (2.5 cells per fibre in untreated progeroids vs. 5.4 in sActRIIB‐treated progeroids after 72 h in culture). Besides direct protective effects on muscle, we show systemic improvements to other organs including the structure and function of the kidneys; there was a major decrease in the protein content in urine (albumin/creatinine of 4.9 sActRIIB treated vs. 15.7 in untreated), which is likely to be a result in the normalization of podocyte foot processes, which constitute the filtration apparatus (glomerular basement membrane thickness reduced from 224 to 177 nm following sActRIIB treatment). Treatment of the progeric mice with the activin ligand trap protected against the development of liver abnormalities including polyploidy (18.3% untreated vs. 8.1% treated) and osteoporosis (trabecular bone volume; 0.30 mm^3^ in treated progeroid mice vs. 0.14 mm^3^ in untreated mice, cortical bone volume; 0.30 mm^3^ in treated progeroid mice vs. 0.22 mm^3^ in untreated mice). The onset of neurological abnormalities was delayed (by ~5 weeks) and their severity reduced, overall sustaining health without affecting lifespan.

**Conclusions:**

This study questions the notion that tissue growth and maintaining tissue function during ageing are incompatible mechanisms. It highlights the need for future investigations to assess the potential of therapies based on myostatin/activin blockade to compress morbidity and promote healthy ageing.

## Introduction

Ageing can be defined as the time‐dependent decline in molecular, cellular, tissue, and organismal function increasing risk for morbidity and mortality. It is the major risk factor for numerous diseases including neurodegeneration, cardiovascular disease, and cancer.^1^ Progress into understanding the mechanisms underlying the ageing process offers the prospect of slowing its progression and maintaining biological systems enabling a healthier life in old age.

Current models of ageing imply interplay between stochastic and genetic components.^2^, ^3^ Random damage in DNA represents a stochastic element. Accumulation of DNA damage‐induced mutations is considered a significant mediator of cancer whereas DNA damage‐induced cellular functional decline, senescence, and death contribute to ageing.^4^ The case for a genetic component comes from numerous studies that have defined the growth hormone/insulin‐like growth factor‐1 (GH/IGF‐1) as a central genetic axis that controls ageing. A spectrum of mutations that attenuate components of the GH/IGF‐1 signalling cascade results in extended lifespan.^5^ The apparently disparate stochastic and genetic components have been reconciled into a unified model of ageing by proposing that accumulation of DNA damage, and thereafter failure of DNA to properly replicate or be transcribed, leads to activation of a survival response programme that attenuates the GH/IGF‐1 activity. The ultimate purpose of dampening GH/IGF‐1 signalling is the prioritization of maintenance mechanisms over those that promote growth.^2^, ^3^, ^6^

Ageing results in the progressive decline of the function of essentially all organ systems. One of the most apparent signs of ageing in humans is sarcopenia, the involuntary loss of skeletal muscle mass and function over time.^7^ It becomes evident at middle age in humans with a loss of 0.5–1% of mass per year, which increases in the seventh decade.^8^ Age‐related muscle loss leads to a disproportionate decrease in strength (1.5–5%/year) relative to the change in its mass, implying a reduction in both the quality and quantity of the tissue.^9^ Sarcopenia invariably leads to a reduced quality of life by impacting on mobility and stability, which leads to increase incidence of fall‐related injury. More importantly, sarcopenia predisposes individuals to adverse disease outcomes (cardiovascular and metabolic diseases) and mortality.^10^, ^11^

Skeletal muscle is a highly adaptable tissue and can be induced to undergo changes in mass as well as composition through numerous interventions including exercise and diet.^12^ Numerous non‐genetic molecular interventions that increase muscle mass have also been designed.^13^, ^14^ One of the most potent reagents is the soluble activin receptor type IIB (sActRIIB) molecule, which acts to neutralize the muscle growth inhibitory properties of myostatin and activin. It induces significant increases in body mass in less than 4 weeks in wild‐type and muscle disease model mice.^15^

A number of investigations using rodents models suggest that maintaining muscle mass and function not only guards against sarcopenia but also promotes longevity, implying that the entire multi‐organ ageing process can be attenuated by such intervention.^16^ However, a mechanism that promotes muscle hypertrophy as an anti‐ageing regime would seemingly conflict with the intended outcome of the adaptive changes mediated through decreased GH/IGF‐1 signalling that focus a body's reserves on tissue maintenance at the expense of growth. Although studies in humans have shown an association between maintaining muscle mass/function and attenuating the impact of sarcopenia (e.g. Duetz *et al*.^17^) and evidence that mass is a predictor for longevity,^10^ there is, to our knowledge, no direct evidence that it directly extends lifespan.

Here, we challenge the notion that tissue growth, specifically in muscle, is incompatible with the systemic maintenance of tissue structure and function during ageing. We have used the progeroid *Ercc1* ^*Δ/−*^ mutant mouse line as an experimental platform for our studies. It harbours attenuated excision repair cross‐complementation 1 activity, a key component of several DNA repair pathways including nucleotide excision repair.^18^ The stochastic increased accumulation of various types of DNA adducts, which normally are repaired by these pathways, explains why ERCC1 mutations in humans cause a complex of clinical features called xeroderma pigmentosum type F‐ERCC1 (XFE) syndrome^2^ combining symptoms of Cockayne Syndrome, a progeroid condition^19^ associated with a transcription‐replication conflicts (TCR) defect as well as Fanconi's anaemia, a cross‐link repair disorder. *Ercc1* ^*Δ/−*^ hypomorphic mutant mice progressively show signs of ageing in all organs from about 8 weeks of age, which are much more severe than in geriatric wild‐type mice^20^, ^21^ (and see Vermeij *et al*. for overview^22^). *Ercc1* ^*Δ/−*^ mutant mice die at 4–6 months of age.^20^, ^23^

Based on the concept that DNA damage induces a survival response that promotes maintenance programmes at the expense of growth, one would predict that augmenting muscle growth would in the long run exacerbate the pathological features in a progeroid model. What we find is something quite different; sActRIIB treatment prior to the onset of progeria can support the growth of skeletal muscle, notwithstanding nucleotide excision repair defects. Importantly, the muscle is free of the numerous ultrastructural abnormalities found in untreated *Ercc1* ^*Δ/−*^ littermates, nor does it build up elevated levels of reactive oxygen species (ROS). We show that these qualitative changes in the muscle are underpinned by an active autophagic programme. At the organismal level, sActRIIB protects *Ercc1* ^*Δ/−*^ mice from age‐related decline in muscle strength and locomotor activity. It also protects kidney function from developing proteinuria, the liver from nuclear abnormalities and metabolic shift, and the skeletal system from osteoporosis and delays the development and severity of neurological abnormalities like tremors. However, lifespan was not increased. We believe that this work highlights the need for future investigations focusing on assessing the therapeutic potential of antagonism of the myostatin/activin signalling cascade in sustaining health and quality of life until old age.

## Methods

### Ethical approval

‘The authors certify that they comply with the ethical guidelines for publishing in the Journal of Cachexia, Sarcopenia and Muscle: update 2017’.^24^ The experiments were performed under a project licence from the United Kingdom Home Office in agreement with the Animals (Scientific Procedures) Act 1986. The University of Reading Animal Care and Ethical Review Committee approved all procedures. Animals were humanely sacrificed via Schedule 1 killing. The Erasmus MC study was in strict accordance with the Guide for the Care and Use of Laboratory Animals of the National Institutes of Health and was approved by the Dutch Ethical Committee (permit # 139‐12‐13), in full accordance with European legislation.

### Animal maintenance

Control (*Ercc1* ^*+/+*^) and transgenic (*Ercc1* ^*Δ/−*^) mice were bred as previously described^20^, ^25^ and maintained in accordance to the Animals (Scientific Procedures) Act 1986 (UK) and approved by the Biological Resource Unit of Reading University or the Dutch Ethical Committee at Erasmus MC. Mice were housed in individual ventilated cages under specific pathogen‐free conditions (20–22°C, 12–12 hr light–dark cycle) and provided food and water *ad libitum*. Because the *Ercc1* ^Δ/−^ mice were smaller, food was administered within the cages, and water bottles with long nozzles were used from around 2 weeks of age. Animals were bred and maintained (for the lifespan cohort) on AIN93G synthetic pellets (Research Diet Services B.V.; gross energy content 4.9 kcal/g dry mass, digestible energy 3.97 kcal/g). Post‐natal myostatin/activin block was induced in 7‐week‐old male mice, through intraperitoneal (IP) injection with 10 mg/kg of sActRIIB‐Fc every week, two times till week 16.^26^ Each experimental group consisted of a minimum of five male mice. The University of Reading experiments were performed on 12 controls, 9 *Ercc1* ^Δ/−^, and 14 sActRIIB‐treated *Ercc1* ^Δ/−^ mice (all male mice). Lifespan experiments were performed on both genders, with five male and five female *Ercc1* ^Δ/−^ mice per treatment condition and four males and four female littermate wild‐type controls. End‐of‐life *Ercc1* ^Δ/−^ animals, both sActRIIB and mock treated, were post‐mortem investigated and scored negative for visible tumours, signs of internal bleedings, enlarged spleen size, or abnormally coloured heart or enlarged heart size.

### Phenotype scoring

The mice were weighed and visually inspected at least weekly and were scored in a blinded manner by experienced research technicians for the onset of various phenotypical parameters. The onset of body weight was counted as the first week. A decline in body weight was noted after their maximal body weight was reached. Whole‐body tremor was scored if mice were trembling for a combined total of at least 10 s when put on a flat surface for 20 s. Impaired balance was determined by observing the mice walking on a flat surface for 20 s. Mice that had difficulties in maintaining an upright orientation during this period were scored as having imbalance. If mice showed a partial loss of function of the hind limbs, they were scored as having paresis.

### Open‐field activity cages monitoring system

Open‐field cages (Linton Instrumentation AM548) with an array of invisible infrared light beams and multiple photocell receptors were used. Beams scan activity at two levels from front to back and left to right was performed to determine movement with data captured using AMON software, running on Windows PCs. The lower grid measured normal X, Y movement, whilst the upper grid measured rearing movement. Mice (14 weeks of age) were acclimatized for 30 min before recording. Data were measured on three occasions at 1 day intervals.

### Rotarod

Rotarod machine (Panlab Harvard Apparatus LE8500; or Ugo Basile for Erasmus MC cohort) was used for motor activity and fatigue characterization. Mice were held manually by the tail and placed on the central rod that rotated at the minimum speed for acclimatization for 1 min. Thereafter, the rotation rate of the central rod was increased to a maximum of 40 rpm. The rotation rate and time mice stayed on the central rod was recorded.

## Grip strength

*In vivo* assessment of forelimb muscle maximum force was performed using a force transducer (Chatillon DFM‐2, Ontario, Canada). Mice were held by the tail base, lowered towards the bar, and allowed to grip. The mouse was pulled backwards, and the force applied to the bar just before loss of grip was recorded.

### Muscle tension measurements

Dissection of the hind limb was carried out under oxygenated Krebs solution (95% O_2_ and 5% CO_2_). Under circulating oxygenated Krebs solution, one end of a silk suture was attached to the distal tendon of the extensor digitorum longus (EDL) and the other to a force transducer (FT03). The proximal tendon remained attached to the tibial bone. The leg was secured in the experimental chamber. Silver electrodes were positioned on either side of the EDL. A constant voltage stimulator was used to directly stimulate the EDL, which was stretched to attain the optimal muscle length to produce maximum twitch tension (*P*_t_). Tetanic contractions were invoked by stimulus trains of 500 ms duration at 20, 50, 100, and 200 Hz. The maximum tetanic tension (*P*_o_) was determined from the plateau of the frequency–tension curve.

### Protein synthesis measure

The relative rate of protein synthesis was measured using the surface sensing of translation method (SUnSET).^27^ Briefly, mice were injected exactly 30 min before tissue collection with 0.04 μmol/g body mass puromycin into the peritoneal cavity and then returned to their cages. After tissue collection, muscles were solubilized as for western blotting and then pulled through a slot blotting chamber facilitating the transfer of protein onto a nylon membrane. Thereafter, the membrane was processed identically to a western blot.

### Histological analysis and immunohistochemistry

Following dissection, the muscle was immediately frozen in liquid nitrogen‐cooled isopentane and mounted in optimal cutting temperature compound (TAAB O023) cooled by dry ice/ethanol. Immunohistochemistry was performed on 10 μm cryosections that were air‐dried at room temperature (RT) for 30 min before the application of block wash buffer [PBS with 5% foetal calf serum (*v*/v), 0.05% Triton X‐100]. Antibodies were diluted in wash buffer 30 min before using. Fluorescence‐based secondary antibodies were used to detect all primary antibodies except for CD‐31 where the Vectastain ABC‐HRP kit was deployed (Vector PK‐6100) with an avidin/biotin‐based peroxidase system and DAB peroxidase (HRP) substrate (Vector SK‐4100). Morphometric analysis of muscle fibre size was performed as previously described.^28^ Details of primary and secondary antibodies are given in *Table* 1.

**Table 1:** Primer and antibody details

Primary antibodies Antigen	Species	Dilution	Supplier
Pax7	Mouse	1:1	DSHB
MyoD	Rabbit	1:200	Santa Cruz Biot # sc‐760
Myogenin	Rabbit	1:200	Santa Cruz sc576
MYHCI	Mouse	1:1	DSHB A4.840
MYHCIIA	Mouse	1:1	DSHB A4.74
MYHCIIB	Mouse	1:1	DSHB BF.F3
CD31	Rat	1:150	AbD serotec MCA2388
Dystrophin	Rabbit	1:200	Abcam 15277
Collagen IV	Rabbit	1:500	Abcam ab6586
Histone H3	Rabbit	1:100	Abcam ab8898
Histone H4	Rabbit	1:200	Abcam ab9052
pSmad2/Smad3	Rabbit	1:200	Cell signalling Technology # 8828
SMA	Mouse	1:300	Sigma A2547
Caspase‐3	Rabbit	1:200	Cell signalling Technology #9664S
Phospho‐S6 Ribosomal Protein (Ser235/236)	Rabbit	1:1000	Cell signalling Technology #4857
Phospho‐Akt (Ser473)	Rabbit	1:1000	Cell signalling Technology #4060
LC3	Rabbit	1:1000	Cell signalling Technology #2775
Phospho‐4E‐BP1 (Thr37/46)	Rabbit	1:1000	Cell signalling Technology #2855
Phospho‐4E‐BP1 (Ser65)	Rabbit	1:1000	Cell signalling Technology #9451
Anti‐p62/SQSTM1	Rabbit	1:1000	Sigma P0067
Phospho‐FoxO1 (Ser256)	Rabbit	1:1000	Cell signalling Technology #9461
Anti‐Smad3 (phospho S423 + S425)	Rabbit	1:200	Abcam (ab52903)
Nephrin	Goat	1:500	R&D Systems (AF3159)
PFoxO3a (Ser253)	Rabbit	1:1000	Cell signalling Technology #9466
Anti‐gamma H2A.X (phospho S139)	Rabbit	1:1000	Abcam 11174

Secondary antibodies			
Antibody	Species	Dilution	Supplier
Alexa fluor 633 anti‐mouse	Goat	1:200	Life Technologies # A20146
Alexa fluor 488 anti‐mouse	Goat	1:200	Life Technologies # A11029
Alexa fluor 488 anti‐rabbit	Goat	1:200	Life Technologies # A11034
Alexa fluor 594 anti‐rabbit	Goat	1:200	Life Technologies # A11037
Immunoglobulins/HRP anti‐Rat	Rabbit	1:200	Dako P0450

qPCR primers sequence	Sequence
Oligo name	
MuRF1.F	ACCTGCTGGTGGAAAACATC
MuRF1.R	CTTCGTGTTCCTTGCACATC
Atrogin.1F	GCAAACACTGCCACATTCTCTC
Atrogin.1R	CTTGAGGGGAAAGTGAGACG
R_mVEGFA189.F	TGCAGGCTGCTGTAACGATG
R_mVEGFA189.R	CTCCAGGATTTAAACCGGGAT T
R_mFGF1.F	GAAGCATGCGGAGAAGAACTG
R_mFGF1.R	CGAGGACCGCGCTTACAG
R_mVEGFB.F	TGCCATGGATAGACGTTTATG C
R_mVEGFB.R	TGCTCAGAGGCACCACCAC
m Ndufb5.F	CTTCGAACTTCCTGCTCCTT
m Ndufb6.R	GGCCCTGAAAAGAACTACG
m Sdha.F	GGAACACTCCAAAAACAGACCT
m Sdha.R	CCACCACTGGGTATTGAGTAGAA
m Sdhc.F	GCTGCGTTCTTGCTGAGACA
m Sdhc.R	ATCTCCTCCTTAGCTGTGGTT
m Cox5b.F	AAGTGCATCTGCTTGTCTCG
m Cox5b.R	GTCTTCCTTGGTGCCTGAAG
m Atp5b.F	GGTTCATCCTGCCAGAGACTA
m Atp5b.R	AATCCCTCATCGAACTGGACG
m Mdh2.F	TTGGGCAACCCCTTTCACTC
m Mdh3.R	GCCTTTCACATTTGCTCTGGTC
m Idh2.F	GGAGAAGCCGGTAGTGGAGAT
m Idh3.R	GGTCTGGTCACGGTTTGGAA
m Idh3a.F	CCCATCCCAGTTTGATGTTC
m Idh3a.R	ACCGATTCAAAGATGGCAAC
R.mPGC1A.F	AACCACACCCACAGGATCAGA
R.mPGC1A.R	TCTTCGCTTTATTGCTCCATGA
m Mvk. F	GGGACGATGTCTTCCTTGAA
m Mvk.R	GAACTTGGTCAGCCTGCTTC
m Srebf1.F	GATCAAAGAGGAGCCAGTGC
m Srebf1.R	TAGATGGTGGCTGCTGAGTG
m Srebf2.F	GGATCCTCCCAAAGAAGGAG
m Srebf2.R	TTCCTCAGAACGCCAGACTT
R_mCD36.F	AGATGACGTGGCAAAGAACAG
R_mCD36.R	CCTTGGCTAGATAACGAACTCTG
R_mSlc25a20.F	CAACCACCAAGTTTGTCTGGA
R_mSlc25a20.R	CCCTCTCTCATAAGAGTCTTCCG
R_mACADL.F	TGCCCTATATTGCGAATTACGG
R_mACADL.R	CTATGGCACCGATACACTTGC
R_mFabp3.F	ACCTGGAAGCTAGTGGACAG
R_mFabp3.R	TGATGGTAGTAGGCTTGGTCAT
R_mDmd.F	ACTCAGCCACCCAAAGACTG(20)
R_mDmd.R	TGTCTGGATAAGTGGTAGCAACA
R_mCol4a1.F	GGCCCCAAAGGTGTTGATG(19)
R_mCol4a1.R	CAGGTAAGCCGTTAAATCCAGG
m Hsp10.F	CTGACAGGTTCAATCTCTCCAC
m Hsp10.R	AGGTGGCATTATGCTTCCAG
m Clpp.F	CACACCAAGCAGAGCCTACA
m Clpp.R	TCCAAGATGCCAAACTCTTG
m IL6.F	GGTGACAACCACGGCCTTCCC
m IL6.R	AAGCCTCCGACTTGTGAAGTGGT
m IL18.F	GTGAACCCCAGACCAGACTG
m IL18.R	CCTGGAACACGTTTCTGAAAGA
m Phb.F	TCGGGAAGGAGTTCACAGAG
m Phb.R	CAGCCTTTTCCACCACAAAT
m Phb2.F	CAAGGACTTCAGCCTCATCC

### Dihydroethidium staining

Sectioned slides were dried for 30 min at RT. The sections were rehydrated with PBS then incubated with dihydroethidium (DHE) (50 μmol/L in PBS Sigma D7008) for 30 min at 37°C in the dark. Counterstain for nuclei was DAPI‐containing fluorescent mounting medium.

### Haematoxylin and eosin

Muscle and liver sections were dewaxed in xylene and rehydration in ethanol prior to incubation with Harris' haematoxylin solution (Sigma HHS16) for 30 s and thereafter in eosin solution (Sigma‐Aldrich 318906) for 2 min.

### Succinate dehydrogenase staining

Muscle cyro‐sections were incubated for 3 min at RT in a sodium phosphate buffer containing 75 mM sodium succinate, 1.1 mM Nitroblue Tetrazolium (Sigma‐Aldrich), and 1.03 mM Phenazine Methosulphate (Sigma‐Aldrich). Samples were then fixed in 10% formal‐calcium, dehydrated and cleared in xylene prior to mounting with DPX mounting medium (Fisher).

### Transmission electron microscopy

Biceps muscle and the kidney were briefly fixed with 4% paraformaldehyde and 2.5% glutaraldehyde in 0.1 M cacodylate buffer pH 7.4 *in situ* at RT then dissected, removed, and cut into pieces of 1 mm^3^ and fixed for 48 h in same solution at 4°C. Tissue blocks were contrasted using 0.5% OsO_4_ (Roth, Germany; RT, 1.5 hr) and 1% uranyl acetate (Polysciences, Germany) in 70% ethanol (RT, 1 hr). After dehydration, tissue blocks were embedded in epoxy resin (Durcopan, Roth, Germany), and ultrathin sections of 40 nm thickness were cut using a Leica UC6 ultramicrotome (Leica, Wetzlar, Germany). Sections were imaged using a Zeiss 906 TEM (Zeiss, Oberkochen, Germany) and analysed using ITEM software (Olympus, Germany).^26^

### Blood glucose, growth hormone, insulin, and insulin‐like growth factor‐1 levels

Glucose levels were measured using a freestyle mini blood glucose metre. GH, insulin, and IGF‐1 levels were measured in serum using a rat/mouse growth hormone ELISA (Merck Millipore), ultrasensitive mouse insulin ELISA (Mercodia), or mouse IGF‐1 ELISA (Abcam), respectively.

### Micro‐computed tomography imaging

Computed tomography imaging was performed using a high‐speed *in vivo* micro‐computed tomography (μCT) scanner (Quantum FX, PerkinElmer, Hopkinton, MA, USA). The X‐ray source was set to a current of 160 μA and a voltage of 90 kVp. The field of view was 30 mm × 30 mm for muscle with a voxel size of 60 μm and 20 mm × 20 mm, and voxel size was 40 μm, for bone. The animals received isoflurane anaesthesia (2.5%) to immobilize them during scanning. Following scanning, image segmentation was performed semi‐automatically using the Volume Edit tools within the analysis software package (AnalyzeDirect, Overland Park, KS, USA). Briefly, segmentation masks (object maps) were created using a combination of semi‐automatic and manual techniques (object extraction, region growing, and thresholding tools). These segmentation results were then manually modified if necessary and quantified using the ROI tools.

### Protein expression by immunoblotting

Frozen muscles were pulverized with pestle and mortar and solubilized in 50 mM Tris, pH 7.5, 150 mM NaCl, 5 mM MgCl_2_, 1 mM DTT, 10% glycerol, 1% SDS, 1% Triton X‐100, 1X Roche Complete Protease Inhibitor Cocktail, and 1X Sigma‐Aldrich Phosphatase Inhibitor Cocktails 1 and 3. Proteins were denatured in Laemmli buffer and resolved on 10% SDS‐PAGEs prior to immunoblotting and probing with antibodies and the SuperSignal West Pico Chemiluminescent substrate (Pierce). Details of antibodies are given in *Table* 1.

### Quantitative polymerase chain reaction

Fifty to 100 mg of tissue was solubilized in TRIzol (Fisher) using a tissue homogenizer. Total RNA was prepared using the RNeasy Mini Kit (Qiagen, Manchester, UK). Five micrograms of RNA were reverse‐transcribed to cDNA with SuperScript II Reverse Transcriptase (Invitrogen) and analysed by quantitative real‐time RT‐PCR on a StepOne Plus cycler, using the Applied Biosystems SYBR‐Green PCR Master Mix. Primers were designed using the software Primer Express 3.0 (Applied Biosystems). Relative expression was calculated using the ΔΔCt method and normalized to cyclophilin‐B and hypoxanthine‐guanine phosphoribosyltransferase. Primer sequences are given in *Table* 1.

### Satellite cell culture

Single fibres from EDL were isolated using 0.2% collagenase I in Dulbecco's modified Eagle's medium and either fixed in 2% paraformaldehyde or cultured for 24, 48, and 72 hr as previously described.^29^

### Bone scanning

Tibial samples were scanned and analysed by μCT; 180° scans were performed on a Skyscan 1172F μCT scanner (Skyscan, Kontich, Belgium); the X‐ray source was operated at 50 kV and 200 uA, a 0.5 aluminium filter was used with a 1650 ms exposure time and a pixel size of 5 μm. Projection images were reconstructed into tomograms using NRecon (Skyscan, Kontich, Belgium), and regions of interest were analysed using CTAn (Skyscan, Kontich, Belgium).

#### Trabecular analysis

The reconstructed datasets were re‐oriented in Dataviewer (Skyscan, Kontich, Belgium) so that the long axis of the bone ran along the Y‐axis, which allowed the tibial length to be measured in CTAn. The reference point for trabecular analysis was the disappearance of primary spongiosa bone and the appearance of the secondary trabecular bone in the centre and subjacent to the epiphyseal growth plate. The volume of interest for trabecular analysis was set as 5% of the tibial length from this reference point down the diaphysis. This volume of trabecular bone was selected using CTAn and then analysed using CTAn BatMan software.

#### Cortical analysis

The reference point for cortical analysis was set as the mid‐point of the diaphysis, and then a volume of interest was selected 0.25 mm in either side of this point, ensuring to remove any trabecular bone within the tomograms. Cortical regions were selected using CTAn and then analysed using CTAn BatMan software.

### Statistical analysis

Data are presented as mean ± SE. Data normal distribution were checked by the D'Agostino‐Pearson omnibus test. Significant differences between two groups were performed by the Student's *t*‐test for independent variables. Differences among groups were analysed by one‐way analysis of variance followed by Bonferroni's multiple comparison tests or the non‐parametric Kruskal–Wallis test followed by the Dunn's multiple comparisons as appropriate. Statistical analysis was performed on GraphPad Prism 5 (La Jolla, USA). Lifespan, onset of neurological phenotypes, and body weight decline were statistically analysed with the survival curve analysis using the product limit method of Kaplan and Meier with Log‐rank Mantel‐Cox test in GraphPad Prism. Differences were considered statistically significant at *P* < 0.05.

## Results

### Characterization of skeletal muscle in the *Ercc1* ^*Δ/−*^ progeroid mouse

We first characterized the muscle phenotype of *Ercc1* ^*Δ/−*^ progeroid mice. It is important to mention that a number of studies have established that the initial development of *Ercc1* ^*Δ/−*^ mice in a uniform FVB/C57Bl6 F1 hybrid genetic background is normal.^20^ After birth, mice are progressively affected leading to accelerated appearance of numerous features of ageing.^22^ Therefore, we decided to investigate muscle from *Ercc1* ^*Δ/−*^ male mice at the age of 16 weeks, when mice show numerous signs of ageing, but before the onset of premature mortality.^20^ At this time, all muscles examined from *Ercc1* ^*Δ/−*^ mice were significantly smaller compared with control animals (ranging from 40% to 60% of normal mass; Supporting Information *Figure* S1A). Surprisingly, even though the muscle mass was decreased, the number of fibres was increased in *Ercc1* ^*Δ/−*^ EDL (significantly) and soleus muscles (not‐significant) (*Figure* S1B). The muscle from the progeric mice had significantly more fibres with centrally located nuclei than controls (*Figure* S1C). Fibre size analysis showed decreases in the cross‐sectional area across most myosin heavy chain (MHC) isoforms in muscles with differing contraction properties [EDL, soleus and tibialis anterior (TA)] of *Ercc1* ^*Δ/−*^ mutants (*Figure* S1D–S1G). There was no evident trend for changes in size in relation to the MHC isoform. Every muscle examined displayed a decrease in the number of fibres expressing the slower forms of MHC and a concomitant increase in the fast fibre population, except for MHCIIa and MHCIIb in the superficial portion of the TA (*Figure* S1H–S1J). We examined the whole muscle for its metabolic status by profiling the proportion of fibres displaying high levels of succinate dehydrogenase (SDH) activity, an indicator of oxidative phosphorylation. These experiments revealed that *Ercc1* ^*Δ/−*^ EDL and soleus muscles contained a lower proportion of oxidative fibres compared with controls (*Figure* S1K).

We next examined features of individual fibres. The number of satellite cells (SC) on EDL fibres from *Ercc1* ^*Δ/−*^ animals was reduced to 50% or less of the normal value (*Figure* S1L). Furthermore, *Ercc1* ^*Δ/−*^ SC were unable to follow the normal proliferation and differentiation programmes and displayed a deficit in the proportion of myogenin‐positive cells and an increase in the number of cells expressing Pax7 (*Figure* S1M). These results show that both the muscle fibre and satellites cells show quantitative and qualitative features associated with extreme ageing.

### The activin ligand trap increases body organismal activity and strength

**Figure 1:**
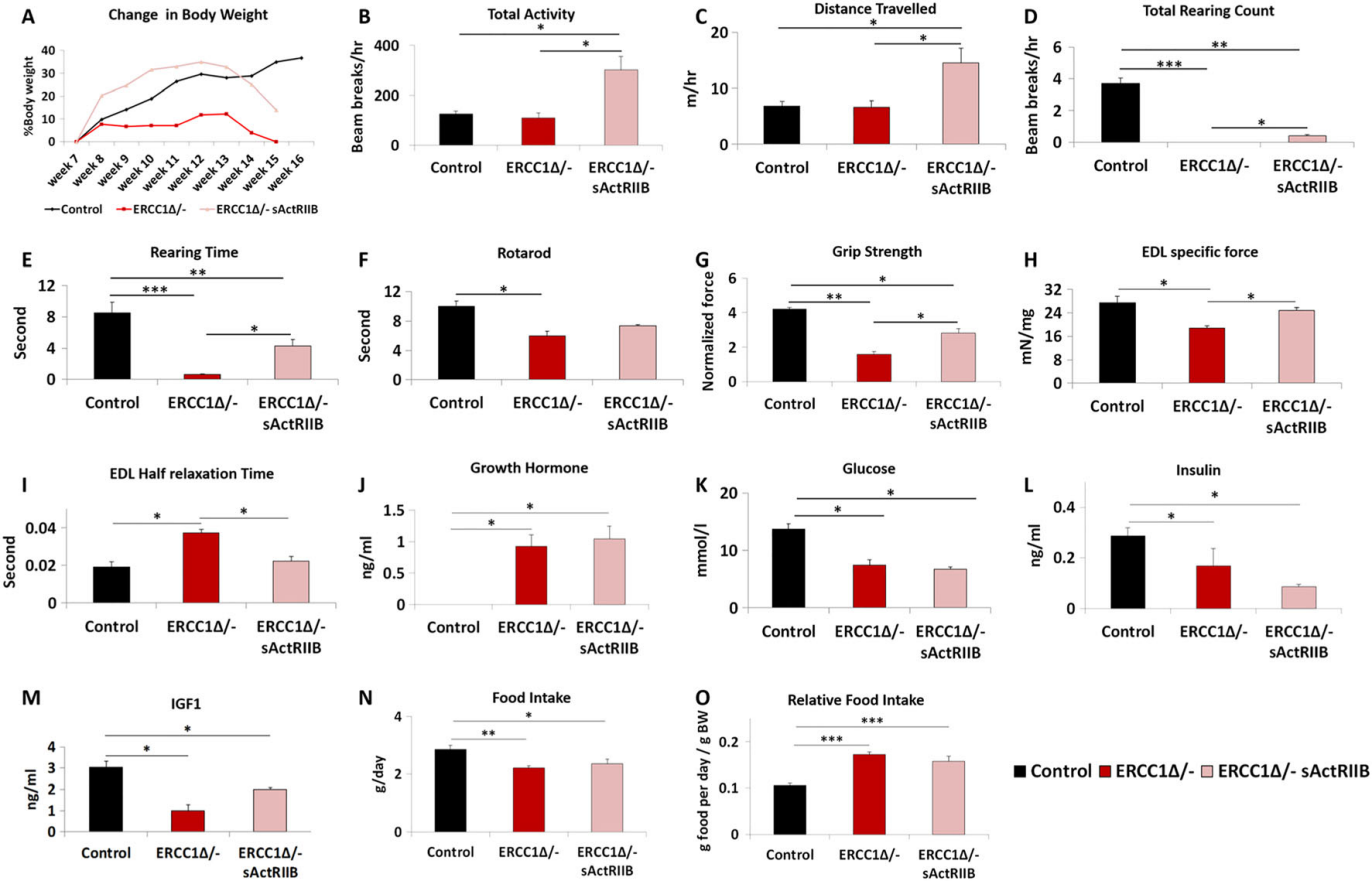
sActRIIB treatment mitigates body, whole animal activity, grip strength, losses, and specific force loss in *Ercc1* ^*Δ/−*^ mice. (A) Relative changes in body mass over time. Intraperitoneal injection of *Ercc1* ^*Δ/−*^ with sActRIIB started at week 7 and tissues collected at the end of week 15. Organismal activity measurements through activity cages. Measurements in (B–E) made at the end of week 14. (F) Rotarod activity. (G) Muscle contraction measurement through assessment of grip strength. (H) *Ex vivo* assessment of EDL‐specific force. (I) Half relaxation time for the EDL. Levels of (J) growth hormone, (K) glucose, (L) insulin, and (M) insulin‐like growth factor‐1 at beginning of week 15. (N) Food intake and (O) relative food intake at the end of week 15. *n* = 6 control male mice, *n* = 5 *Ercc1* ^*Δ/−*^ untreated male mice, and *n* = 5 *Ercc1* ^*Δ/−*^ treated male mice. All analysis performed using non‐parametric Kruskal–Wallis test followed by the Dunn's multiple comparisons except (J) where one‐way analysis of variance followed by Bonferroni's multiple comparison tests was used. *P < 0.05, **P < 0.01, ***P < 0.001. EDL, extensor digitorum longus; IGF‐1, insulin‐like growth factor‐1; sActRIIB, soluble activin receptor type IIB.

We determined whether the age‐related reduced muscle mass in *Ercc1* ^*Δ/−*^ mutants could be prevented by the sActRIIB protein, which we have shown to antagonize signalling mediated by myostatin and related proteins.^26^ To that end, male *Ercc1* ^*Δ/*−^ mice were IP injected twice a week with sActRIIB from 7 weeks of age till week 16. Mock‐treated *Ercc1* ^*Δ/−*^ mutants showed no overall body mass gain in 8 weeks, whereas both control and *Ercc1* ^*Δ/−*^ animals treated with sActRIIB displayed weight increases of 37% and 18%, respectively (*Figures* 1A, S2A, and S2B).

Using activity cages, we found that sActRIIB‐treated *Ercc1* ^*Δ/−*^ mice were more active than both their mock‐treated counterparts and control mice (*Figure* 1B and *Movie* S1). Treatment of *Ercc1* ^*Δ/−*^ mice with sActRIIB increased the distance travelled compared not only with untreated mice but also with control animals (*Figure* 1C and *Movie* S1). Total rearing counts and rearing time, measures of locomotor activity as well as exploration and anxiety, were highest in control mice and significantly reduced in *Ercc1* ^*Δ/−*^ mice. sActRIIB treatment increased these values compared with *Ercc1* ^*Δ/−*^ but not to normal levels (*Figure* 1D–1E). Motor coordination, measured using the Rotarod, showed that *Ercc1* ^*Δ/−*^ mice at the age of 16 weeks have significant deficit in this skill, which was improved, albeit not to normal levels, by sActRIIB (*Figure* 1F). Muscle function, as assessed using a grip metre, revealed that progeric mice had reduced strength compared with control mice. This parameter was significantly improved in *Ercc1* ^*Δ/−*^ mutants by sActRIIB (*Figure* 1G). *Ex vivo* measure of specific force revealed a significant deficit in this parameter in *Ercc1* ^*Δ/−*^ mutants that was significantly increased by sActRIIB treatment (*Figure* 1H). Half‐relaxation time was increased in *Ercc1* ^*Δ/−*^ mutants compared with controls but reduced by sActRIIB treatment (*Figure* 1I).

We determined the circulatory levels of molecules known to regulate organismal growth and found elevated levels of GH in both untreated and sActRIIB treated *Ercc1* ^*Δ/−*^ mutants (*Figure* 1J), likely as previously noted feedback mechanism in response to prolonged low IGF‐1.^2^ Indeed, levels of blood glucose, serum insulin, and IGF‐1 were decreased in *Ercc1* ^*Δ/−*^ mutants as compared with controls, and none of these factors were changed in response to sActRIIB treatment (*Figure* 1K–1M). Food intake of *Ercc1* ^*Δ/−*^ mutants, relative to body weight, was higher than control mice, but unaffected by sActRIIB treatment, excluding indirect effects of diet restriction for which *Ercc1* ^*Δ/−*^ mice are very sensitive (*Figure* 1N–1O).^23^ Water intake was not affected by the treatment (data not shown).

### Quantitative and qualitative improvements to skeletal muscle through soluble activin receptor type IIB treatment

**Figure 2:**
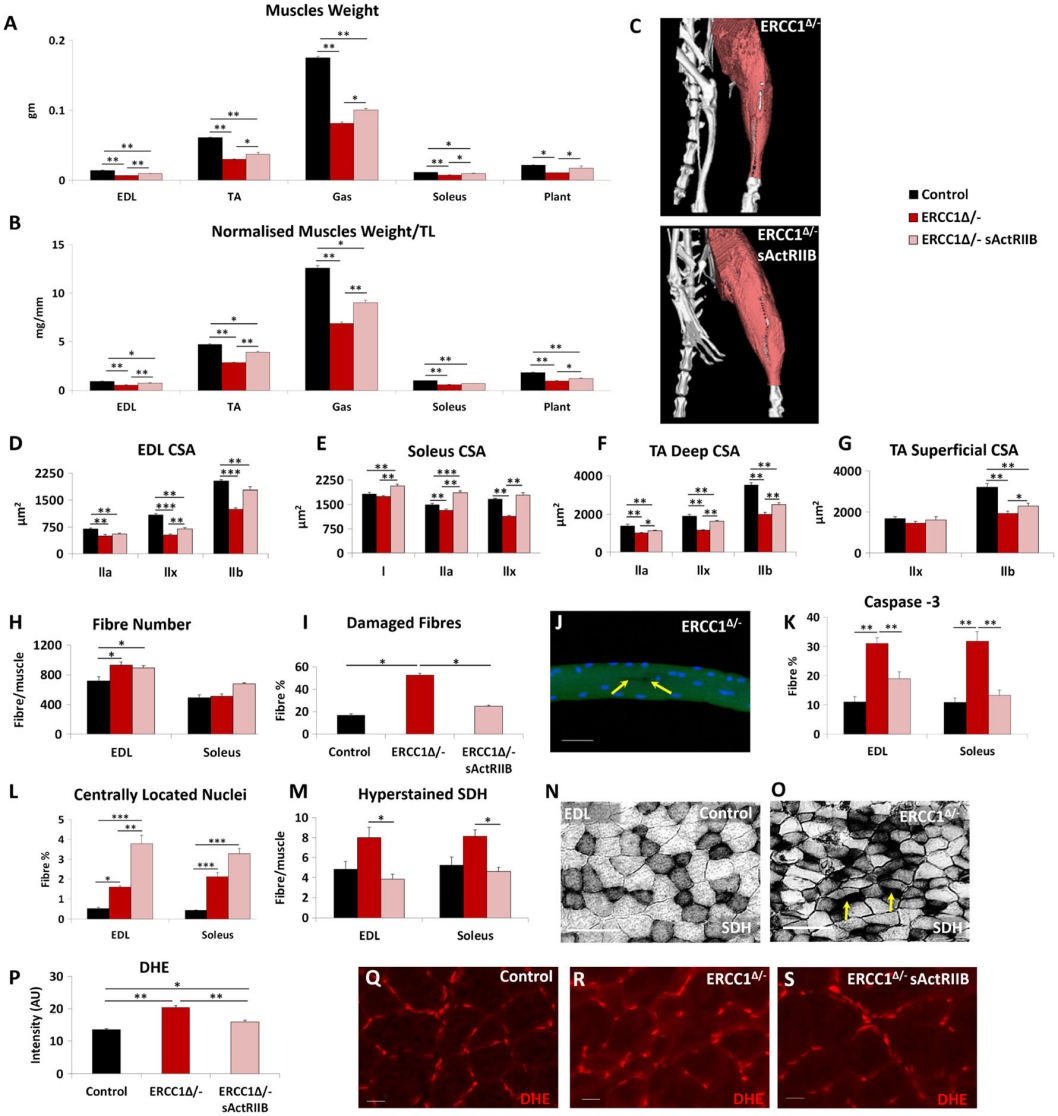
Quantitative and qualitative improvements to *Ercc1* ^*Δ/−*^ skeletal muscle through sActRIIB treatment. (A) Muscle weight at end of week 15. (B) Muscle mass normalized to tibial length. (C) Micro‐computed tomography scan of hind limb to visualize the increase in muscle upon sActRIIB treatment in *Ercc1* ^*Δ/−*^ mice. (D–G) Cross‐sectional fibre areas assigned to specific myosin heavy chain isoforms. (H) Fibre number increased in EDL and soleus of *Ercc1* ^*Δ/−*^ mice and further increased following treatment. (I) Incidence of damaged fibres following single fibre isolation. (J) Example of micro‐tear (arrows) in an *Ercc1* ^*Δ/−*^ EDL fibre. (K) Fibres containing caspase 3 epitope as a percentage of all EDL and soleus fibres. (L) Percentage of fibres with centrally located nuclei in the EDL and soleus. (M) Quantification of hyper‐stained SDH fibres. (N) SDH in control muscle and (O) *Ercc1* ^*Δ/−*^ muscle showing hyper‐stained fibres (arrows). (P) Quantification of DHE fluorescence in TA muscle fibres. (Q) Control TA fibres with little DHE fluorescence in the body of control fibres. (R) *Ercc1* ^*Δ/−*^ TA fibres with elevated DHE fluorescence in the body of control fibres. (S) Treated *Ercc1* ^*Δ/−*^ TA fibres with little DHE fluorescence in the body of fibres. *n* = 9 control male mice, *n* = 8 *Ercc1* ^*Δ/−*^ untreated male mice, and *n* = 8 *Ercc1* ^*Δ/−*^ treated male mice. Scale for single fibre 50 μm, SDH 100 μm and DHE 20 μm. One‐way analysis of variance followed by Bonferroni's multiple comparison tests. *P < 0.05, **P < 0.01, ***P < 0.001. DHE, dihydroethidium; EDL, extensor digitorum longus; sActRIIB, soluble activin receptor type IIB; SDH, succinate dehydrogenase; TA, tibialis anterior.

Previous work has shown that sActRIIB treatment increases muscle mass. The increased body weight and grip strength of *Ercc1* ^*Δ/−*^ mice subjected to sActRIIB prompted us to further examine individual muscles. Treated *Ercc1* ^*Δ/−*^ mice revealed that all five groups showed significant greater mass compared with those from mock‐treated *Ercc1* ^*Δ/−*^ animals with a range of 30–62% (TA and plantaris, respectively; *Figure* 2A–2C). Activation of signalling pathways initiated through ActRIIB and relevant to this study was found to be elevated in the muscle of *Ercc1* ^*Δ/−*^ mice and decreased by sActRIIB treatment (*Figure* S3A). Importantly, the abundance of DNA breaks was not changed by sActRIIB treatment (*Figure* S3B). Furthermore, sActRIIB failed to increase the mass of any other organ examined including the heart, kidney, and liver (*Figure* S2C and S2D). We explored the mechanisms underlying the increase in muscle mass following sActRIIB treatment of *Ercc1* ^*Δ/−*^ mice. Introduction of sActRIIB induced fibre hypertrophy irrespective of MHC expression (*Figure* 2D–2G). Of particular note was the finding that some types of fibres in the sActRIIB‐treated *Ercc1* ^*Δ/−*^ muscles were significantly larger than even in controls (see MHCI and IIA in soleus; *Figure* 2E). The total fibre number in EDL was elevated in *Ercc1* ^*Δ/−*^ mutants and maintained by sActRIIB (*Figure* 2H). A similar trend was found in the soleus (*Figure* 2H). Of particular note was the observation of a large proportion of fibres with micro‐lesions (including tears to the membrane) isolated from the EDL muscle from *Ercc1* ^*Δ/−*^ animals, which appeared largely normalized by sActRIIB (*Figure* 2I and 2J). Caspase‐3 activity as a gauge of apoptosis was significantly elevated in muscle of *Ercc1* ^*Δ/−*^ mice and largely normalized by treatment with sActRIIB (*Figures* 2K and S3C). The number of fibres displaying centrally located nuclei was elevated in both the EDL and soleus muscles from *Ercc1* ^*Δ/−*^ mice compared with controls and became even more abundant following sActRIIB treatment (*Figures* 2L and S3D). The fibres showing supra‐normal levels of SDH activity, indicative of abnormal mitochondrial activity that leads to apoptosis,^30^ were significantly more frequent in both the EDL and soleus of *Ercc1* ^*Δ/*−^ mice compared with treated mutants (*Figure* 2M–2O). Assessment of ROS activity through DHE intensity showed elevated levels of superoxide in muscle of *Ercc1* ^*Δ/−*^ animals, which was lowered by sActRIIB treatment although it did not reach the level of control mice (*Figure* 2P–2S).^31^, ^32^

### Myosin heavy chain, oxidative fibre profiling, vascular organization, and molecular metabolic analysis of skeletal muscle

**Figure 3:**
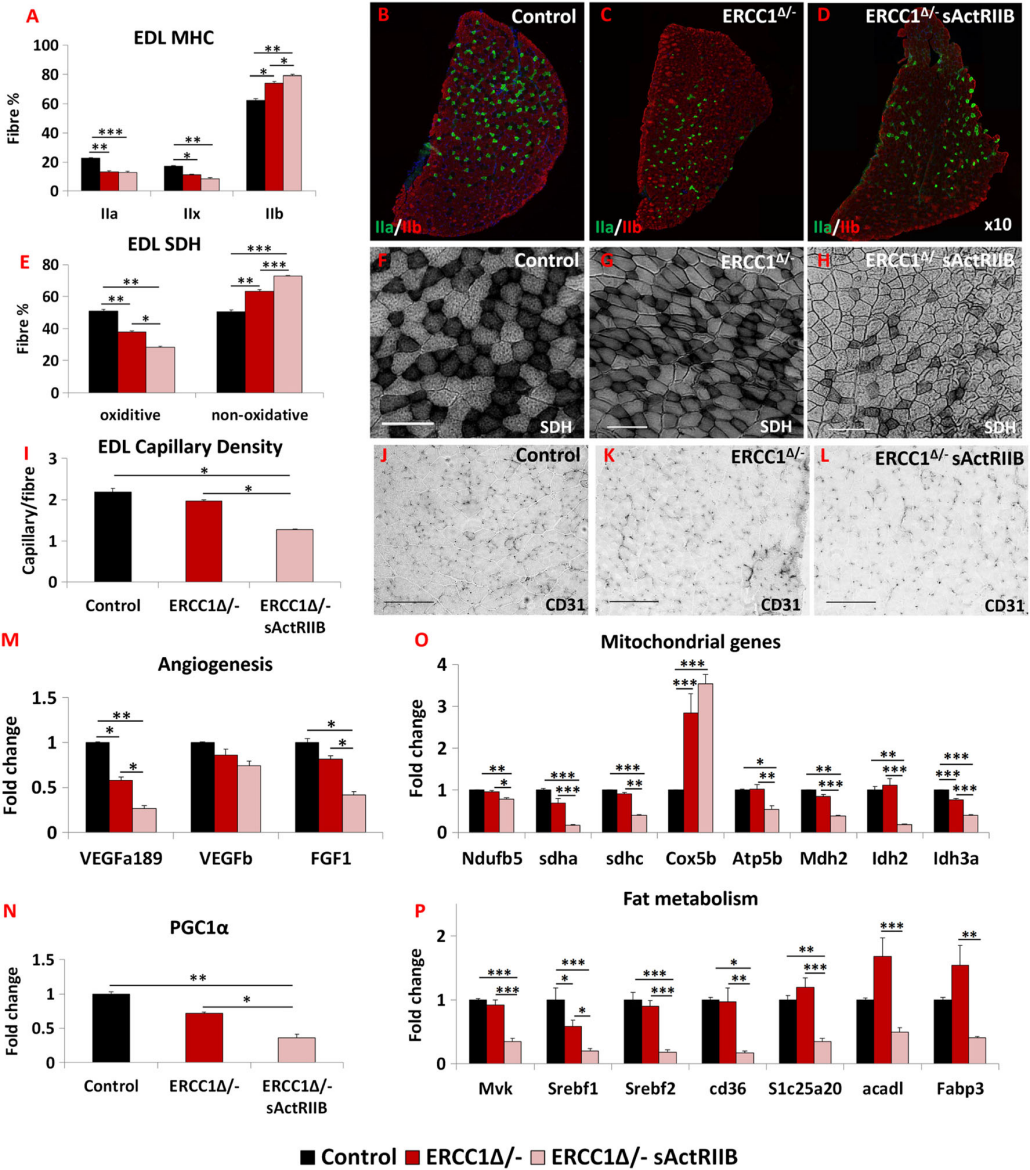
sActRIIB induces fast and glycolytic transformation of *Ercc1* ^*Δ/−*^ muscle. (A) MHC profile of EDL muscle. (B–D) EDL MHCIIA/IIB fibre distribution in the three cohorts, controls, *Ercc1* ^*Δ/−*^, and *Ercc1* ^*Δ/−*^ treated with sActRIIB. (E) SDH‐positive and ‐negative fibre profile of EDL muscle. (F–H) SDH stain in the three cohorts. (I) Quantification of EDL capillary density. (J–L) Identification of EDL capillaries with CD‐31 in the three cohorts. Quantitative PCR profiling of (M) angiogenic genes, (N) PGC1α, (O) mitochondrial genes, and (P) regulators of fat metabolism. *n* = 8 for all cohorts. Scale for SDH 100 μm and CD31 50 μm. One‐way analysis of variance followed by Bonferroni's multiple comparison tests used in all data sets except (E) where non‐parametric Kruskal–Wallis test followed by the Dunn's multiple comparison was used. *P < 0.05, **P < 0.01, ***P < 0.001. EDL, extensor digitorum longus; MHC, myosin heavy chain; SDH, succinate dehydrogenase; sActRIIB, soluble activin receptor type IIB.

Myosin heavy chain analysis revealed that the progeric muscle displayed a faster profile compared with control muscles (*Figure* S1H–S1J). Treatment of progeric animals with sActRIIB resulted in a shift towards an even faster MHC profile. This was particularly pronounced in the EDL, with an increase in the proportion of type IIB fibres at the expense of both types IIA and IIX (*Figure* 3A–3D).

To examine the metabolic status of the sActRIIB‐treated muscle, we determined the SDH activity. In both the EDL and the soleus, the number of SDH‐positive fibres was decreased in the progeric mice compared with controls (*Figures* 3E–3H and S3E). Introduction of sActRIIB treatment further decreased the number of SDH^+^ fibres and, at the same time, increased the number of SDH^−^ entities in the EDL (*Figure* 3E–3H). Similar changes were also recorded in the soleus (*Figure* S3E). Therefore, the sActRIIB treatment further reduces the status of the already diminished oxidative character of *Ercc1* ^*Δ/−*^ muscles. Subsequently, we investigated whether changes in the muscle metabolic profile wrought by sActRIIB also induced a remodelling of the vasculature. The capillary density profile indeed showed that the number of blood vessels serving each fibre was lower in *Ercc1* ^*Δ/−*^ mice (albeit non‐significantly) and further decreased following sActRIIB treatment (*Figure* 3I–3L). These changes were underpinned by decreases in the expression of three genes examined that control the development of blood vessels (*Figure* 3M). Expression of *PGC1α*, a key regulator of oxidative properties in muscle, was slightly lower in muscle from *Ercc1* ^*Δ/−*^ mice compared with controls and was even more suppressed following sActRIIB treatment (*Figure* 3N). The changes in genes supporting the development of blood vessels were mirrored by mitochondrial transcript levels. qPCR analysis of eight genes important for the mitochondrial metabolism revealed that seven had decreased expression in *Ercc1* ^*Δ/−*^ muscles induced by sActRIIB treatment (*Figure* 3O). We also investigated genes that control fat metabolism. All seven genes examined were significantly reduced in expression by sActRIIB (*Figure* 3P).

Therefore, the attenuation of signalling through sActRIIB results in the patterning of muscle towards a fast contracting status, which has a paucity of oxidative fibres and supporting blood vessels underpinned by changes in the expression of genes that control capillary development and sustain aerobic metabolism.

### Ultrastructure and mitochondrial characterization in muscle

**Figure 4:**
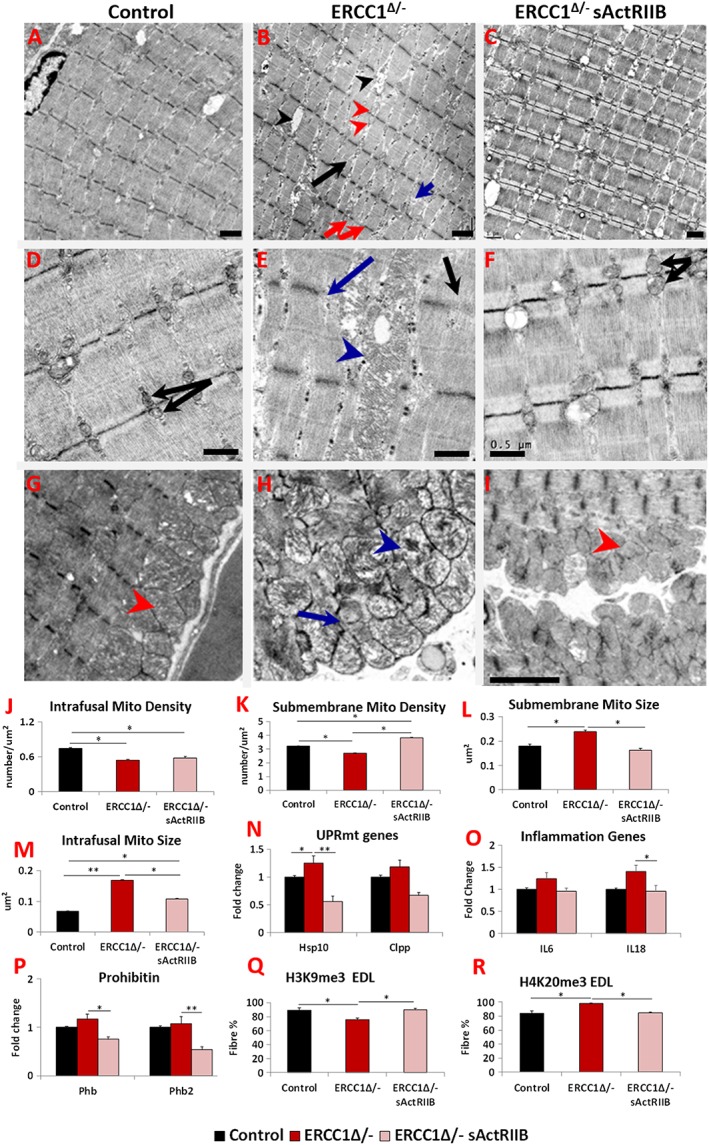
sActRIIB prevents *Ercc1* ^*Δ/−*^ muscle ultrastructural abnormalities and supports normal levels of expression of key stress indicators. All Electron microscopy (EM) longitudinal image and quantitative measurements are from the bicep muscle. (A) Low‐power image of control muscle. (B) Low‐power image of *Ercc1* ^*Δ/−*^ muscle. Note large spaces (black arrowheads), non‐uniform sarcomere width (red arrows), dilated sarcomeric mitochondria (red arrowheads), split sarcomere (black arrow), and disrupted M‐Line (blue arrow). (C) Low‐power image of sActRIIB‐treated *Ercc1* ^*Δ/−*^ muscle. (D) Higher magnification of sarcomeric region of control muscle showing uniformly sized mitochondria (black arrows). (E) Enlarged mitochondria in sarcomeric region of *Ercc1* ^*Δ/−*^ muscle (blue arrowhead) and absent (blue arrow) or faint Z‐line (black arrow). (F) Higher magnification of sarcomeric region of treated *Ercc1* ^*Δ/−*^ mice showing smaller sarcomeric mitochondria (black arrows). (G) Sarcolemma region of control muscle showing compact mitochondria (red arrowhead). (H) Dilated (blue arrowhead) and aberrant mitochondria (blue arrow) in sub‐sarcolemma region of *Ercc1* ^*Δ/−*^ muscle. (I) Sarcolemma region of treated *Ercc1* ^*Δ/−*^ mice showing compact mitochondria (red arrowhead). (J, K) Sarcomeric (intrafusal) and sub‐membrane mitochondrial density measurements. (L, M) Sub‐membrane and sarcomeric (intrafusal) mitochondrial size measurements. (N) Expression of mitochondria unfolded protein response gene in gastrocnemius muscle. (O) Expression of inflammatory genes in gastrocnemius muscle. (P) Expression of *prohibitin* genes in gastrocnemius muscle. (Q) Quantification of EDL fibres expressing H3K9me3 and (R) H4K20me3. EM studies *n* = 6–7 for all cohorts. All other measures *n* = 8–9 for all cohorts. Non‐parametric Kruskal–Wallis test followed by the Dunn's multiple comparisons used in (N, O) and the rest with one‐way analysis of variance followed by Bonferroni's multiple comparison tests. *P < 0.05, **P < 0.01. EDL, extensor digitorum longus; sActRIIB, soluble activin receptor type IIB.

The ultrastructure of skeletal muscle in the three cohorts was examined using transmission electron microscopy. Numerous abnormalities were evident in the muscle from *Ercc1* ^*Δ/−*^ mice including heterogeneous Z‐line lengths, missing Z‐lines, misaligned Z‐lines, split sarcomeres, and large inter‐sarcomeric spaces compared with controls (*Figure* 4A, 4B, 4D, 4E, 4G, and 4H). These abnormalities were largely absent in muscle from *Ercc1* ^*Δ/−*^ mice treated with sActRIIB (*Figure* 4C, 4F, and 4I). Quantification of mitochondria density revealed a decrease in this parameter both within the fibre (sarcomeric region) and directly under the sarcolemma (*Figure* 4J and 4K). Of special note was the alteration (swelling) of mitochondria both within the fibre and immediately under the sarcolemma (*Figure* 4H). Quantification of mitochondrial size showed enlargement in the muscle from *Ercc1* ^*Δ/−*^ mutants, which was reduced by the treatment with sActRIIB (*Figure* 4L and 4M). Mitochondrial hypertrophy has been shown to be a protective response to a decrease in mitochondrial function or number or an indicative excessive fusion.^33^, ^34^, ^35^ It is thought to promote mitochondrial survival by up‐regulating a stress response programme. Indeed, we found that there was an increase in the expression of key genes involved in the mitochondrial unfolded protein response (UPR^MT^) pathway in the muscle from *Ercc1* ^*Δ/−*^ mice (*Figure* 4N). We also examined the levels of inflammatory and prohibitin genes, which support mitochondrial function of ensuring correct folding of the cristae.^36^ Expression of *IL6* and *IL18* as well as two key prohibitin genes (*Phb* and *Phb2*) appeared slightly elevated in the muscle of *Ercc1* ^*Δ/−*^ mice (*Figure* 4O and 4P). Treatment of *Ercc1* ^*Δ/−*^ mice with sActRIIB generally prevented these changes (*Figure* 4A–4P). Lastly, we examined whether muscle harboured epigenetic modifications involved in the maintenance of heterochromatin that change with age.^37^, ^38^ The ageing process causes a decrease in the level of H3K9me3 but an increase in H4K20me3.^39^ H3K9me3 was decreased, and H4K20me3 increased in *Ercc1* ^*Δ/−*^ animals in keeping with an age‐related change (*Figure* 4Q and 4R). Both features were normalized following sActRIIB treatment (*Figure* 4Q and 4R).

These results demonstrate subcellular defects in the *Ercc1* ^*Δ/−*^ muscle and the expression of genes indicative of ongoing stress. sActRIIB treatment prevented the development of many of these abnormal features.

### Connective tissue profiling

**Figure 5:**
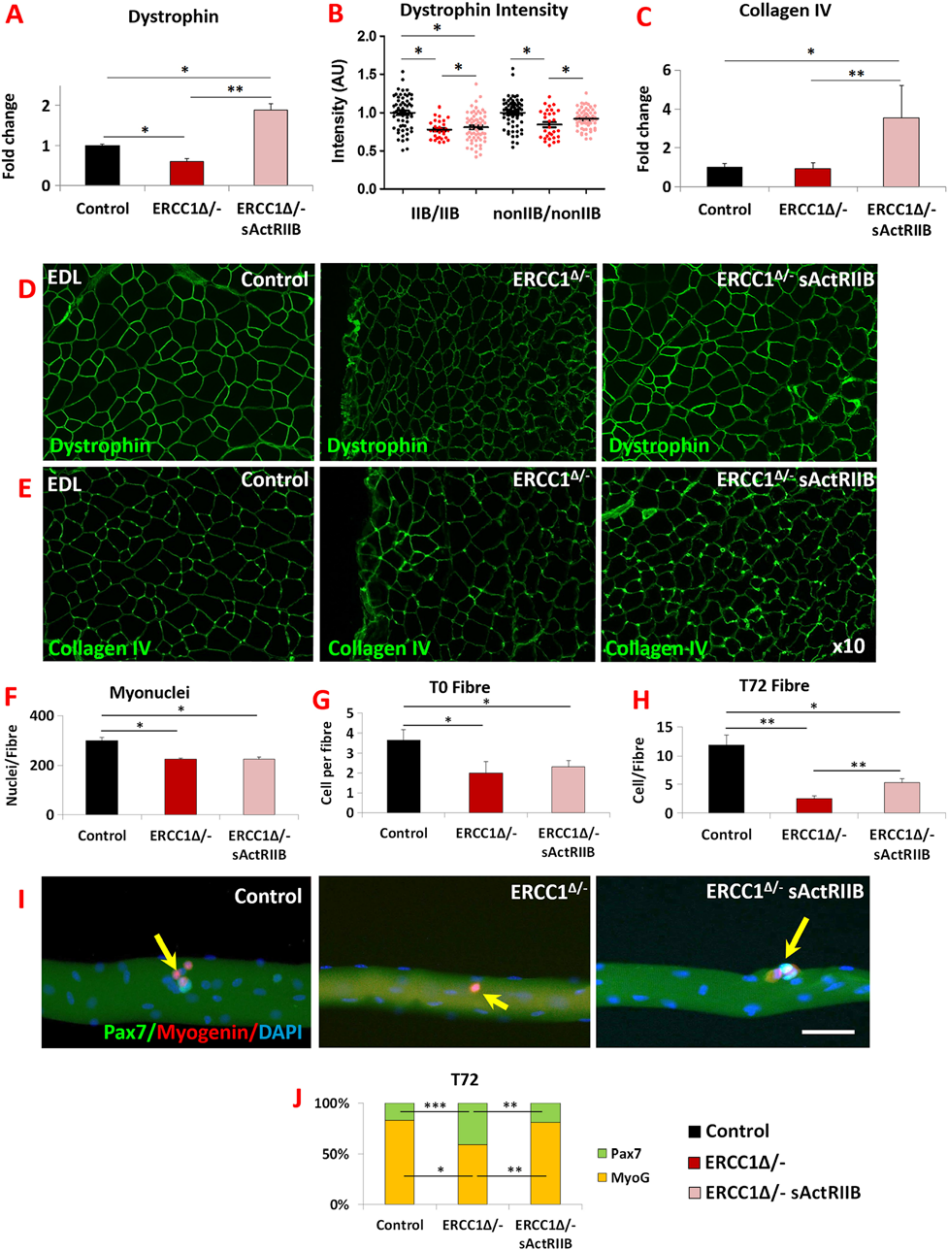
Normalization of *Ercc1* ^*Δ/−*^ extracellular components by sActRIIB and differentiation and self‐renewal of its satellite cells. (A) *Dystrophin* gene expression measured by quantitative PCR (qPCR). (B) Measure of dystrophin in fibre‐type‐specific manner using quantitative immunofluorescence. (C) Measure of *collagen IV* expression profiling by qPCR. (D) Immunofluorescence image for dystrophin expression in EDL muscle. (E) Immunofluorescence image for collagen IV expression in EDL muscle. *n* = 7 for all cohorts. (F) EDL myonuclei count. (G) Quantification of satellite cells on freshly isolated EDL fibres. (H) Quantification of cells on EDL fibres after 72 h culture. (I) Control, mock‐treated *Ercc1* ^*Δ/−*^, and sActRIIB‐treated *Ercc1* ^*Δ/−*^ fibre examined at 72 h for expression of Myogenin (red) and Pax7 (green). Arrows indicated satellite cell progeny. (J) Quantification of EDL differentiated (Pax7^−^/Myogenin^+^) vs. stem cell (Pax7^+^/Myogenin^−^) after 72 h in culture. Fibres collected from three mice from each cohort and minimum of 25 fibres examined. Scale 50 μm. Non‐parametric Kruskal–Wallis test followed by the Dunn's multiple comparisons used for (A–C). Rest of data was analysed using one‐way analysis of variance followed by Bonferroni's multiple comparison tests. *P < 0.05, **P < 0.01, ***P < 0.001. EDL, extensor digitorum longus; sActRIIB, soluble activin receptor type IIB.

Skeletal muscle force transmission relies on proteins that link the contractile apparatus to the extra cellular matrix (ECM). We examined two of its components and determined how they were modified by the *Ercc1* ^*Δ/−*^ genotype and thereafter by sActRIIB treatment. First, we examined the expression of *dystrophin*, a key intercellular component that links the cytoskeleton to the ECM. Its RNA expression was decreased in the *Ercc1* ^*Δ/−*^ muscle, which was subsequently increased to levels greater than controls by sActRIIB (*Figure* 5A). We measured the amount of dystrophin specifically located between fibres using quantitative immuno‐fluorescence and confirmed its reduction specifically at this site in the *Ercc1* ^*Δ/−*^ muscle compared with controls and was significantly increased by sActRIIB (*Figure* 5B and 5D). Thereafter, we examined expression of *collagen IV* as basement membrane component important for force transmission. Its expression was slightly decreased albeit not reaching statistical significance in *Ercc1* ^*Δ/−*^ muscle (*Figure* 5C). However, sActRIIB caused its level to increase over both untreated *Ercc1* ^*Δ/−*^ and control levels (*Figure* 5C). Collagen IV gene expression levels were reflected at the protein level at the myofibre surface (*Figure* 5E).

### Mechanisms underlying fibre size changes

To explore mechanisms regulating muscle mass, we investigated changes in anabolic and catabolic programmes. Surprisingly, levels of phosphorylated Akt (an inducer of anabolism) appeared elevated in the muscle from 16‐week‐old mock‐treated *Ercc1* ^*Δ/−*^ mice (*Figure* S4A). Next, we examined downstream targets of pAkt and found that there was a slight decrease in the phosphorylation of 4EBP1 at Thr37/46 but none at Ser65 (*Figure* S4B). However, there was an elevated level of phosphorylation at another pAkt target, S6 (*Figure* S4C). The effect of sActRIIB on the anabolic programme of *Ercc1* ^*Δ/−*^ muscle showed a general increase in the level of pAkt, as well as its two downstream targets, 4EBP1 and S6, relative to both mock‐treated *Ercc1* ^*Δ/−*^ and control groups (*Figure* S4A–S4C). Thereafter, we probed the catabolic programme and found that activity of FoxO1 and FoxO3a, key regulators of both ubiquitin‐mediated protein breakdown (FoxO1 significantly, FoxO3a not so), was generally decreased in the muscle from *Ercc1* ^*Δ/−*^ mice (*Figure* S4D and S4E), even to a level exceeding controls. Expression of both *MuRF1* and *Atrogin‐1* downstream targets of FoxO1 and FoxO3a were elevated at the RNA level in the muscle of *Ercc1* ^*Δ/−*^ mice (*Figure* S4I and S4J). The LC3 autophagy activity was suppressed compared with controls (*Figure* S4F). Treatment with sActRIIB caused an elevation in the levels of inactive FoxO1 and FoxO3a (*Figure* S4D and S4E) and a decrease in the expression of *MuRF1* but, surprisingly, not *Atrogin‐1* (*Figure* S4I and S4J). Expression of *Mul1*, a key regulator of mitophagy,^40^ did not differ in the three groups (*Figure* S4K). Significantly, we found an increase in the level of autophagy gauged by the LC3II/I ratio and levels of p62 following sActRIIB treatment (*Figure* S4F and S4G). We quantified the presence of p62 puncta, which has been used as an indicator of autophagic flux, with an increase in the numbers of p62 puncta implying a decrease in autophagic activity.^41^ The number of p62 puncta per given area were higher in *Ercc1* ^*Δ/−*^ EDL muscle compared with controls, and their levels were reduced by sActRIIB treatment (*Figure* S4L and S4M). Treatment of *Ercc1* ^*Δ/−*^ mice with sActRIIB resulted in a non‐significant increase in the amount of active eIF2a, a key regulator of the endoplasmic reticulum UPR (UPR^ER^) programme (*Figure* S4H). At the organismal level, we found that the rate of protein synthesis was elevated (but not to significant levels) in *Ercc1* ^*Δ/−*^ mice and further elevated by sActRIIB treatment (*Figure* S4N). The abundance of ubiquitinated proteins was elevated in the muscle of *Ercc1* ^*Δ/−*^ mice but reduced by sActRIIB treatment (*Figure* S4O).

These results reveal novel characteristics considering the changes in muscle mass in the progeric mice. The muscle of *Ercc1* ^*Δ/−*^ mice activates both its protein synthesis pathway and has elevated gene expression of molecules that control protein breakdown. However, autophagy is blunted. Treatment of *Ercc1* ^*Δ/−*^ with sActRIIB results in an increase in the activity of molecules controlling protein synthesis as well as overall rate of protein synthesis, a decrease in the abundance of ubiquitinated proteins E3 as well as an increase in key regulators of autophagy.

### Myonuclei and satellite cell profiling

We examined features of individual myofibres to determine the effect of sActRIIB treatment. The number of myonuclei in the fibres from the EDL or the number of SC on them from either PBS‐ or sActRIIB‐treated *Ercc1* ^*Δ/−*^ mutants was significantly lower than the number in control mice (*Figure* 5F and 5G). We then investigated the proliferative capacity of the SC from the three cohorts and found that, after 72 h of culture, the population from control fibres had undergone a three‐fold increase compared with initial numbers. In sharp contrast, the SC from PBS‐treated *Ercc1* ^*Δ/−*^ mice failed to undergo any significant proliferation. Importantly, sActRIIB treatment of *Ercc1* ^*Δ/−*^ mice resulted in SC being able to undergo a 2.3‐fold increase in number (*Figure* 5H). Finally, we found that the attenuated differentiation programme of SC from *Ercc1* ^*Δ/−*^ mice was normalized by sActRIIB treatment (*Figure* 5I–5J).

Therefore, sActRIIB treatment mitigates abnormalities in SC proliferation, differentiation and self‐renewal programmes in *Ercc1* ^*Δ/−*^ animals. However, it did not normalize the low SC number found in mock‐treated *Ercc1* ^*Δ/−*^ mice.

### Inhibition of glomerular anomalies in *Ercc1* ^*Δ/−*^ mice by soluble activin receptor type IIB

**Figure 6:**
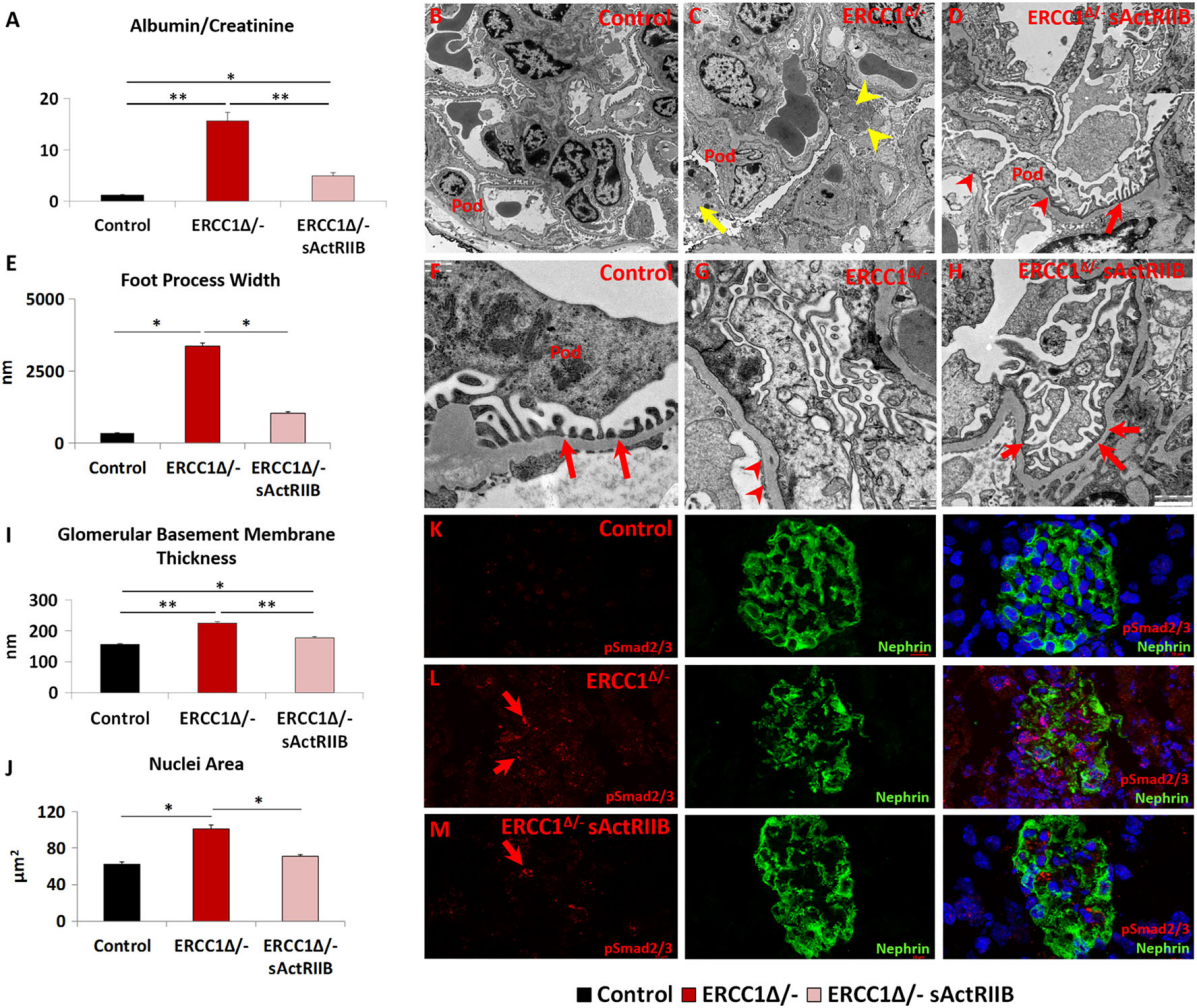
The prevention of kidney function abnormalities through the maintenance of the filtration barriers by sActRIIB treatment of *Ercc1* ^*Δ/−*^ mice. (A) Urine protein measurements at the end of week 14. (B–D) Low and (F–H) high magnification of electron microscopy images of podocytes from control, mock‐treated *Ercc1* ^*Δ/−*^, and sActRIIB‐treated *Ercc1* ^*Δ/*−^ mice. Pod indicates the podocyte. (C) *Ercc1* ^*Δ/−*^ tissue contains autophagosomes (yellow arrow) and enlarged mitochondria (yellow arrowhead). (D) sActRIIB‐treated *Ercc1* ^*Δ/−*^ mice show some foot process effacement (red arrowheads) but significant number of mature foot processes (red arrow). (E) Quantification of foot process width. (F) Numerous mature foot processes in control sample (red arrows). (G) Very few foot processes in *Ercc1* ^*Δ/−*^ sample but thickened glomerular basement membrane (red arrowheads). (H) Treated *Ercc1* ^*Δ/−*^ sample showing numerous mature foot processes (red arrows). (I) Quantification of glomerular basement membrane thickness. (J) Nuclear size measurements in Nephrin‐positive domain. (K) pSmad2/3 profile in control mice (red) in relation to podocytes, identified through Nephrin expression. (L) Abundant levels of pSmad2/3 (red arrows) in *Ercc1* ^*Δ/−*^ podocytes. (M) Few pSmad2/3 puncta in sActRIIB‐treated *Ercc1* ^*Δ/−*^ podocytes (red arrow). *n* = 8 mice examined for each cohort for (A) and *n* = 5 mice examined for each cohort for (EM). Analysis performed using non‐parametric Kruskal–Wallis test followed by the Dunn's multiple comparisons. *P < 0.05, **P < 0.01. sActRIIB, soluble activin receptor type IIB.

Kidney pathology due to mutations in *ERCC1* has been reported in both human and mice.^2^, ^20^ Here, we investigated the impact of sActRIIB on kidney function and structure. Proteinuria analysis showed a 12‐fold elevation in the albumin/creatinine ratio in urine from *Ercc1* ^*Δ/−*^ mutants compared with controls at 16 weeks of age. This measure was reduced to an elevation of 3.7‐fold in the urine of sActRIIB‐treated *Ercc1* ^*Δ/−*^ mice (*Figure* 6A). We investigated the mechanism underlying the proteinuria in *Ercc1* ^*Δ/−*^ mice and how it is influenced by sActRIIB by examining the ultrastructure of the kidney filtration apparatus. Transmission electron microscopy showed the *Ercc1* ^*Δ/−*^ podocytes hypertrophic, but additionally, they contained numerous abnormalities, including enlarged mitochondria as well as accumulation of autophagosomes (*Figure* 6B, 6C, 6F, and 6G, autophagosomes shown in detail in *Figure* S5A). The most prominent feature was the degree of foot process effacement in the *Ercc1* ^*Δ/−*^ sample, which contrasted the regular structures found in control samples (*Figure* 6E–6G). When foot processes were present, they are significantly broader in *Ercc1* ^*Δ/−*^ animals compared with controls (*Figure* 6E–6G). Glomerular basement membrane was also significantly thicker in *Ercc1* ^*Δ/−*^ kidneys compared with controls (*Figure* 6F, 6G, and I6). All these features were to a greater degree normalized following the treatment with sActRIIB (*Figure* 6B–6I). At the ultrastructural level, enlarged mitochondria area and accumulation of autophagosomes were completely prevented (*Figure* 6D and 6H). Foot processes were evident (*Figure* 6H). It should be noted that in some regions, they appeared normal, whereas in other regions, they are still broader compared with controls (*Figure* 6D and 6H). The thickness of the glomerular basement membrane was significantly reduced by sActRIIB treatment compared with mock‐treated progeroid mice but not to normal levels (*Figure* 6I). Nuclear size that was enlarged in the glomeruli of *Ercc1* ^*Δ/−*^ specimens was maintained at normal dimensions by sActRIIB (*Figure* 6J). Finally, we examined whether the impact of sActRIIB could be through direct antagonism of myostatin/activin signalling by investigating the distribution of pSmad2/3 in podocytes. There was very little pSmad2/3 in control glomeruli (*Figure* 6K). In contrast, abundant pSmad2/3 was found in nuclei of *Ercc1* ^*Δ/−*^ podocytes (*Figure* 6L). Following sActRIIB treatment, the abundance of pSmad2/3 in *Ercc1* ^*Δ/−*^ podocytes was reduced compared with untreated progeroid mice (*Figure* 6M). However, it was still more prominent than controls.

These results show that foot process effacement is an underlying cause of proteinuria in *Ercc1* ^*Δ/*^kidneys. sActRIIB treatment not only improved the ultrastructure of the filtration barrier but significantly also reduced proteinuria.

### Impact of soluble activin receptor type IIB on ageing‐related liver *Ercc1* ^*Δ/−*^ abnormalities

**Figure 7:**
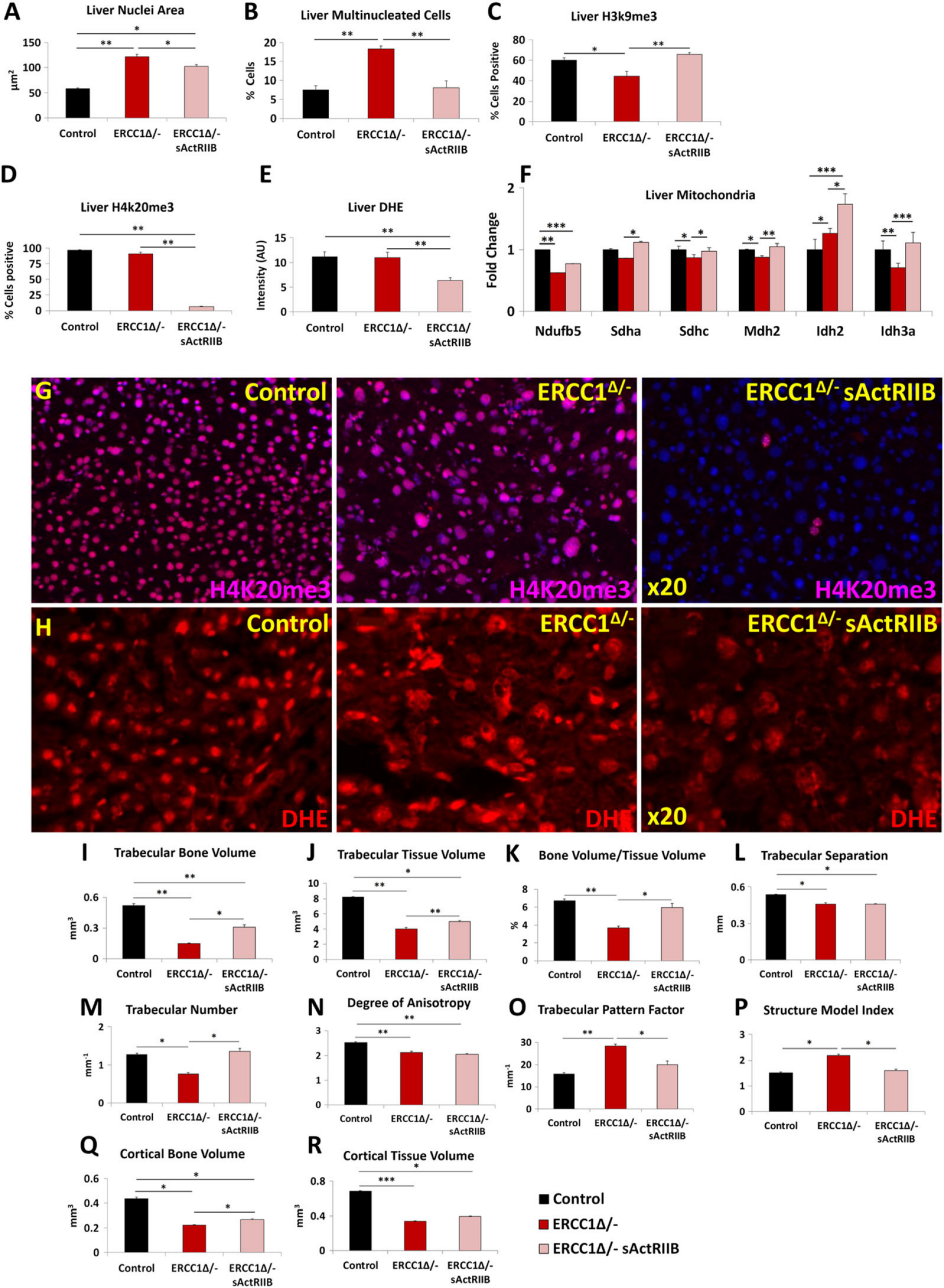
sActRIIB prevents the development age‐related liver abnormalities and osteoporotic phenotype in *Ercc1* ^*Δ/−*^. (A) Measure of liver nuclear size. (B) Profile frequency of multinucleated liver cells. (C) Frequency of H3K9me3‐positive liver cells. (D) Frequency of H4K20me3‐positive liver cells. (E) Quantification of DHE fluorescence to gauge superoxide levels. (F) Quantitative PCR profiling of mitochondrial gene expression. (G) Immunofluorescence images for H4K20me3 distribution in the three cohorts. (H) DHE intensity levels in the three cohorts. (I) Trabecular bone volume measurements. (J) Trabecular tissue volume measurements. (K) Trabecular bone to tissue volume ratios. (L) Trabecular separation indices. (M) Enumeration of trabeculae. (N) Degrees of trabecular anisotrophy. (O) Trabecular pattern factor as a quantification of bone architecture. (P) Structure model index. (Q) Measure of cortical bone volume. (R) Cortical tissue volume measure. Trabecular bone volume measurements. *n* = 8 for all animals in (A–H) and *n* = 6 control male mice, five *Ercc1* ^*Δ/−*^‐untreated male mice, and six *Ercc1* ^*Δ/−*^‐treated male mice in other experiments. One‐way analysis of variance followed by Bonferroni's multiple comparison tests used for (A–F) and non‐parametric Kruskal–Wallis test followed by the Dunn's multiple comparisons for (I–R). *P < 0.05, **P < 0.01, ***P < 0.001. DHE, dihydroethidium; sActRIIB, soluble activin receptor type IIB.

The liver undergoes age‐related changes both in humans and rodent models.^21^, ^42^, ^43^ The nuclei in the livers of *Ercc1* ^*Δ/−*^ mice undergo progressive ageing‐related changes including enlargement, invaginations, and polyploidy. These features have been interpreted to indicate incomplete cytokinesis.^44^ We found that both liver nuclear size and the number of liver multi‐nucleated cells were increased in tissue from *Ercc1* ^*Δ/*−^ mice compared with control tissue (*Figure* 7A and 7B). Treatment of *Ercc1* ^*Δ/−*^ mice with sActRIIB significantly decreased both measures (*Figure* 7A and 7B). Having shown that age‐associated changes in the liver nuclei of *Ercc1* ^*Δ/−*^ mice were reduced following treatment with sActRIIB, we examined whether this was reflected by changes in epigenetic modification involved in the maintenance of heterochromatin.^37^, ^38^ Previous work has shown that levels of H3K9me3 are down‐regulated during ageing,^39^ and here too, we found such a relationship (*Figure* 7C). In contrast, ageing results in an increase in H4K20me3 marks. Here, we saw extensive levels of H4K20me3 in the liver of *Ercc1* ^*Δ/−*^ and surprisingly of control mice (*Figure* 7D and 7G). Strikingly, the H4K20me3 marks were essentially absent in livers of *Ercc1* ^*Δ/−*^ mice treated with sActRIIB (*Figure* 7D and 7G). Oxidative stress is one of the key drivers that induce age‐related changes in the liver.^45^ Again, we deployed the DHE dye to gauge the level of superoxide.^31^, ^32^ Superoxide levels were elevated in the liver samples of both *Ercc1* ^*Δ/−*^ and control mice, compared with treated *Ercc1* ^*Δ/−*^ mice (*Figure* 7E and 7H). Next, we profiled the metabolic activity of the liver as it is known to undergo a decrease in the level of oxidative phosphorylation with ageing.^21^, ^46^ In agreement with the work by Gregg *et al*. on *Ercc1* ^*Δ/−*^ livers, we found a decrease in four of the six genes linked to oxidative phosphorylation (*Figure* 7F).^21^ Expression of five genes was significantly increased by sActRIIB treatment relative to their levels in mock *Ercc1* ^*Δ/−*^ animals (*Figure* 7F). Lastly, we determined whether the effects of sActRIIB on the livers of *Ercc1* ^*Δ/−*^ mice were mediated by direct antagonism of myostatin/activin signalling. Profiling of pSmad2/3 showed that there was no activity in the parenchyma of the livers of the three cohorts (*Figure* S5B). Only a few pSmad2/3‐expressing cells were found adjacent to smooth muscle in all three cohorts (*Figure* S5B).

These results show that antagonism of myostatin/activin signalling leads to profound normalization of *Ercc1* ^*Δ/−*^ liver cell nuclear structure, selective epigenetic modification of DNA and changes in gene expression indicative of increased oxidative phosphorylation and a reduction in superoxide levels.

### Prevention of the osteoporotic phenotype in *Ercc1* ^*Δ/−*^ mice by soluble activin receptor type IIB

Micro‐CT analyses revealed that *Ercc1* ^*Δ/−*^ mice exhibit a premature ageing‐related osteoporotic phenotype with extreme differences in trabecular and cortical bone mass and architecture compared with control mice. In the trabecular compartment, there was a significant reduction in bone volume, tissue volume, bone volume/tissue volume, trabecular separation, trabecular number, and degree of anisotropy, a measure of how highly oriented substructures are within a volume (*Figure* 7I–7N). Significantly higher trabecular pattern factor indicating trabecular connectivity and structure model index a measure of surface convex curvature and an important parameter in measuring the transition of osteoporotic trabecular bone from a plate‐like to rod‐like architecture were also observed in *Ercc1* ^*Δ/−*^ mice compared with controls animals (*Figure* 7O). Cortical bone volume and tissue volume were significantly lower, further demonstrating that *Ercc1* ^*Δ/−*^ mice have an osteoporotic bone phenotype (*Figure* 7Q and 7R).

Analysis of trabecular bone revealed significant increase in bone and tissue volume, bone/tissue volume, and trabecular number in *Ercc1* ^*Δ/−*^ sActRIIB‐treated mice compared with mock‐treated animals, indicating treatment prevents a decrease in the size of the trabecular compartment and the amount of bone present (*Figure* 7I–7K). In addition, trabecular pattern factor was significantly lower in the treated group compared with mock‐treated with levels close to the control group, showing trabecular connectivity improved upon treatment (*Figure* 7O). Furthermore, the structure model index was significantly lower in the treated group, again with results close to the control group (*Figure* 7P). Cortical bone volume and tissue volume were significantly lower in *Ercc1* ^*Δ/−*^ mice compared with the control groups, with treatment significantly lessened the tissue volume and bone volume decline (*Figure* 7Q and 7R).

Together, these analyses reveal that sActRIIB treatment produces tibial architecture changes and prevents a decrease in trabecular and cortical bone mass in *Ercc1* ^*Δ/−*^ mice, mitigating the premature ageing‐related osteoporotic phenotype observed in this and previous studies.

### Long‐term effects of soluble activin receptor type IIB administration to *Ercc1* ^*Δ/−*^ mice

**Figure 8:**
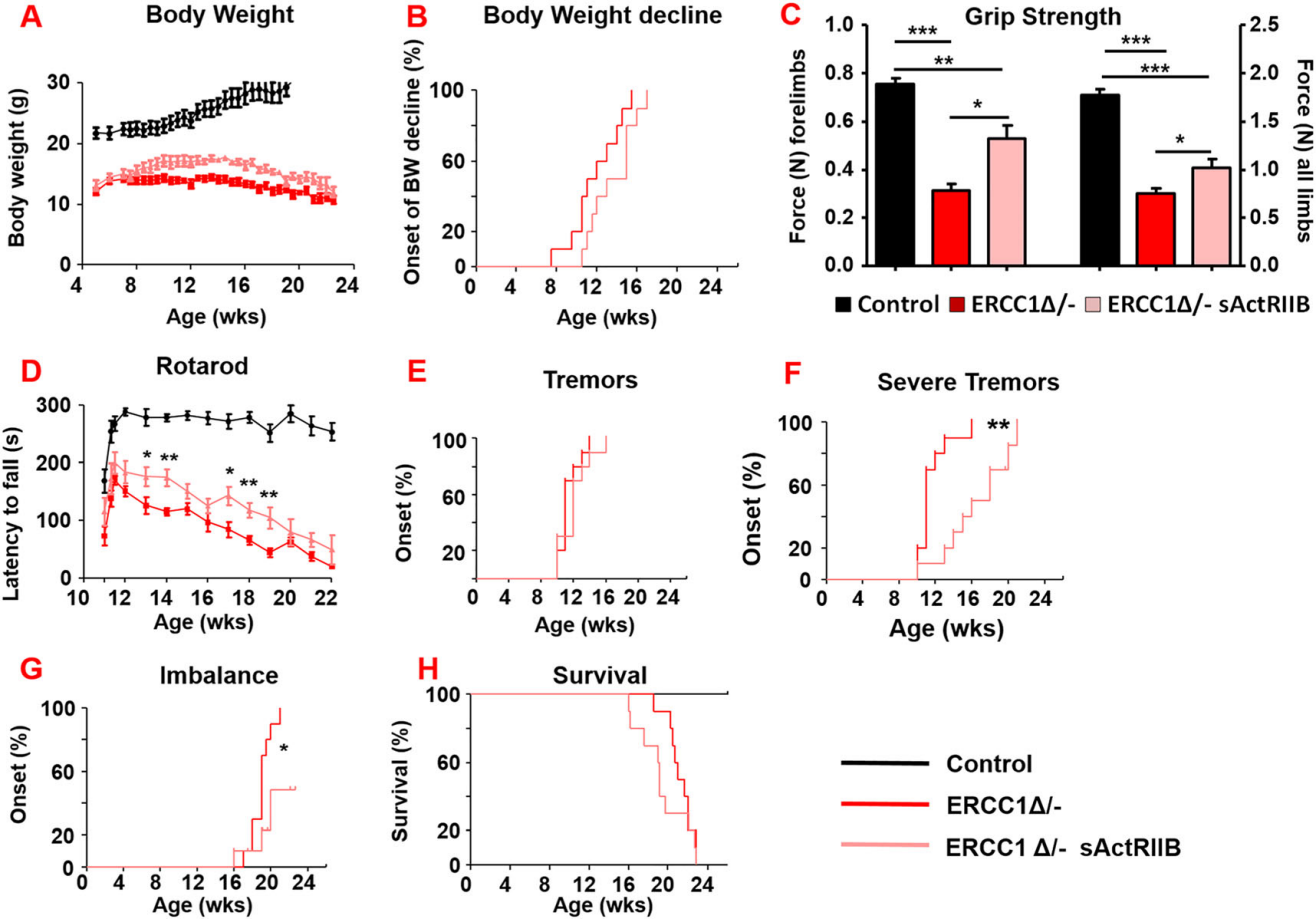
sActRIIB delays neurological abnormalities in *Ercc1* ^*Δ/−*^ mice without affecting lifespan. (A, B) Body weight changes of treated (sActRIIB or mock control) *Ercc1* ^*Δ/−*^ mice at a second test site (*P* = 0.07). Intraperitoneal injection started at week 7. (C) Average grip strength of the forelimbs and all limbs of 4‐month‐old *Ercc1* ^*Δ/−*^ mice under mock and sActRIIB conditions. (D) Average time spent on an accelerating rotarod of *Ercc1* ^*Δ/−*^ mice on different treatments weekly monitored. (E–G) Onset of neurological abnormalities (F) tremors (*P* = 0.28), (F) severe tremors (*P* = 0.0014), and (G) imbalance (*P* = 0.021) with age. (H) Survival of sActRIIB‐treated and mock‐treated *Ercc1* ^*Δ/−*^ mice (*P* = 0.27). *n* = 10 animals per group. Error bars indicate mean ± SE. Log‐rank Mantel‐Cox test. *P < 0.05, **P < 0.01, ***P < 0.001. sActRIIB, soluble activin receptor type IIB.

To confirm the previous results and monitor phenotypical age‐related changes beyond the age investigated so far, we initiated a second cohort of *Ercc1* ^*Δ/−*^ mice at another location. Treatment regime, regarding timing, dosage, and frequency, was kept identical. Similarly, *Ercc1* ^*Δ/−*^ mice reached a higher body weight upon IP injection of sActRIIB as compared with PBS‐injected mutant mice (*Figure* 8A). No gender‐specific response was found in terms of body weight changes due to sActRIIB treatment of *Ercc1* ^*Δ/−*^ mice (*Figure* S6B). As a consequence of the ageing‐associated deterioration, they all gradually declined with age after reaching their maximal body weight, which was delayed by sActRIIB administration (*Figure* 8B). Simultaneously, *in vivo* imaging showed a substantial increase in both muscle and bone volume (*Figure* S6A) confirming the robustness of sActRIIB treatment.

All animals from the sActRIIB group had a more vigorous and lively appearance and showed an improved grip strength for both the forelimbs and all limbs (*Figure* 8C). Additionally, locomotor function, as measured by Rotarod performance, was significantly improved by sActRIIB over the entire lifespan, but still declined with age parallel to the mock‐treated mice (*Figure* 8D).

A prominent ageing feature of these mice is related to neurodegeneration and the onset of several neuro‐muscular phenotypic changes.^22^ Longitudinal examination of behavioural abnormalities showed that the onset of tremors was not delayed following sActRIIB treatment but was reduced in severity (*Figures* 8E, 8F, and S6C and *Table* 2). The onset of imbalance was greatly postponed and frequently absent as well as the onset of paresis of the hind legs (*Figure* 8G and *Table* 2). Nevertheless, sActRIIB treatment of *Ercc1* ^*Δ/−*^ mice did not extend survival of the animals (*Figures* 8H and S6D). These results show that attenuating myostatin/activin signalling prolongs health span rather than delaying death.

**Table 2:** sActRIIB administration attenuates progeroid phenotypes of *Ercc1* ^*Δ*/−^ mice

Symptoms	Age at onset (weeks)		Change of onset (weeks)	# of Ercc1^Δ/−^ mice (Mock, sActRIIB)
	Mock	sActRIIB		
Clasping	5.00	4.80	−0.20	(10, 10)
Tremors	11.40	12.10	**0.70**	(10, 10)
Severe tremors	11.60	16.11	**4.51**	**(10, 9)**
Body weight decline	11.80	13.60	**1.80**	(10, 10)
Kyphosis	17.75	18.15	0.40	(10, 10)
Imbalance	18.95	18.33	−0.62	**(10, 3)**
Paresis	20.50	18.80	−1.67	**(6, 3)**

The average age at onset of characteristic progeroid phenotypes in treated *Ercc1* ^*Δ*/−^ mice and the difference between the group averages is shown. The last column indicates the total number of mice out of 10 per group that displays the phenotype before end of life. Phenotypes delayed more than 0.5 weeks on average or absent in mice treated with sActRIIB compared with mock treated *Ercc1* ^*Δ*/−^ mice are indicated in bold. sActRIIB, soluble activin receptor type IIB.

## Discussion

The key findings of this study are, first, that the sarcopenic programme in the *Ercc1* ^*Δ/−*^ progeroid mouse model not only shows many parallels with naturally aged rodent muscle but also reaches more severe stages and displays several distinctive features. Second, we demonstrate that sarcopenia was attenuated through the antagonism of myostatin/activin signalling despite persistent defective DNA repair. Third, we reveal that inhibition of myostatin/activin signalling induces multi‐systemic physiological improvements; mice increased locomotor activity; increased specific force and kidney function; improved key features of liver biology; mitigated the osteoporotic phenotype; and delayed parameters of neurodegeneration.

*Ercc1* ^*Δ/−*^ muscle parallels with natural muscle ageing and pathological muscle diseases.

We first defined the characteristics of muscle in the *Ercc1* ^*Δ/−*^ progeroid model in light of previous work and discovered many unexpected features related to muscle composition rather than in its overall mass. At the quantitative level, all muscle groups from *Ercc1* ^*Δ/−*^ mice were much lighter than control mice, which concords with findings in aged humans and mouse models.^47^, ^48^ In addition, our studies revealed numerous qualitative differences between progeroid muscle and muscle of aged wild‐type mice. All MHC fibre types were smaller in *Ercc1* ^*Δ/−*^ muscle, and most had undergone a slow to fast fibre profile shift. These features differ from wild‐type mouse muscle where MHCIIB preferentially undergo aged‐related atrophy,^49^ and fibres in both humans and rodents undergo a shift from fast to slow.^50^ We also discovered that the number of fibres in both EDL and soleus muscles were higher than controls, which seems counter‐intuitive given the overall loss in muscle mass in *Ercc1* ^*Δ/−*^ mice. Parallels are invited between the ostensible hyperplasia in our sarcopenic condition and the increased fibre numbers seen in myo‐pathological conditions, such as Duchenne muscular dystrophy.^51^ We suggest that the increased fibre number is due to the abundance of split fibres, a notion supported by our results showing that muscle from *Ercc1* ^*Δ/−*^ mutants contains a large proportion of damaged fibres. Additional findings including the high level of caspase activity and the decrease in dystrophin and collagen lead us to propose that the muscle fibres from *Ercc1* ^*Δ/−*^ mice have elevated levels of contraction‐induced damage which leads to cellular lesions (splitting) and ultimately results in fibre death. Fibre apoptosis and necrosis are a common feature of age‐related muscle wasting but not of disuse atrophy. Indeed, we found that the proportion of dying fibres was far greater in *Ercc1* ^*Δ/−*^ muscle than found in aged wild‐type mice.^30^

The muscle of *Ercc1* ^*Δ/−*^ mice contains numerous abnormal fibres (identified through central nucleation) and dying fibres, but those that seem normal contain subcellular aberrations. Ultrastructural examination reveals abnormalities in the organization of the contractile apparatus and the cellular organelles. Of note, mitochondria were quantitatively and qualitatively affected by the *Ercc1* mutation in muscle. Their density was decreased both within the fibre as well as under the sarcolemma where they would support contraction and membrane‐related activities, respectively.^52^ Furthermore *Ercc1* ^*Δ/−*^ muscle mitochondria at both sites were swollen indicative of response to either functional deficit or altered fusion.^33^, ^34^ Our studies show elevated levels of ROS through the profiling of DHE activity.^53^ We therefore suggest that the *Ercc1* mutation in muscle leads to ultimately compromised mitochondrial function, resulting in increased ROS production, which may compromise the function of the contractile apparatus as well as ultimately inducing fibre death.^52^

Analysis of key proteins involved in anabolic and catabolic programmes revealed an interesting landscape. Surprisingly, *Ercc1* ^*Δ/−*^ muscle showed elevated levels of Akt activity, one of two downstream genes (S6) and overall rate of protein synthesis. The muscle of *Ercc1* ^*Δ/−*^ mice expressed high levels of *MuRF1* and *Atrogin‐1* and contained increased levels of ubiquitinated proteins. Hence, the muscle of *Ercc1* ^*Δ/−*^ animals had initiated a programme of protein synthesis yet, at the same time, was promoting their breakdown, which significantly deviates from normal catabolic conditions.^54^ Although unusual in the context of normal physiology, these results agree with other studies of progeroid models that demonstrate the activation of pathways to limit the effect of the primary lesion.^21^ We suggest that in the context of *Ercc1* ^*Δ/−*^ muscle, the activation of the protein synthesis pathway acts to decrease an extremely high rate of muscle wasting. Nevertheless, the ultimate deregulation of protein synthesis, proteasome and autophagy pathways in *Ercc1* ^*Δ/−*^ muscle, which parallels many disease conditions, culminates in atrophy.^55^, ^56^

We also found changes in the number and behaviour of SC of the *Ercc1* ^*Δ/−*^ muscle; not only were they fewer in number but they also displayed an inability to divide following the isolation of single muscle fibres as well as an attenuated ability to differentiate. Some but not all these features are shared by SC from sarcopenic human muscle; SC from sarcopenic human muscle were shown to be more prone to activation but not follow through a normal degree of differentiation.^57^ However, comparisons of outcomes from different studies are problematic due to use of differing experimental systems.

### Effects of soluble activin receptor type IIB on *Ercc1* ^*Δ/−*^ muscle phenotype

Our study documents the profound effect of antagonizing myostatin/activin signalling on body and muscle mass as well as function of *Ercc1* ^*Δ/−*^ mice. Age‐related decreases in all three parameters were significantly attenuated in the *Ercc1* ^*Δ/−*^ mutant by the soluble activin receptor ligand trap. The difference in the muscle mass between treated vs. untreated animals ranged between 30% (TA) and 62% (plantaris). The changes in muscle mass are extremely impressive and worthy of comparison with the *Mstn* ^*−/−*^ mutant. The EDL of the *Mstn* ^*−/−*^ was 60% heavier than the wild‐type counterpart.^58^ Here, after only 8 weeks of sActRIIB treatment, the EDL was 45% heavier. The differences in muscle mass between treated and untreated mice are of note when compared with outcomes that have previously deployed anti‐myostatin/activin, non‐genetic approaches in aged mice. One such study showed that the TA of aged mice underwent an increase of 6%, whereas here, even though it was the muscle that displayed the smallest increase nevertheless was 30% heavier than that of mock‐treated *Ercc1* ^*Δ/−*^ mice.^59^ These results suggest that activity of myostatin and/or activin in *Ercc1* ^*Δ/−*^ mice are considerably higher than in aged wild‐type mice. Importantly, we show that treatment with sActRIIB did not induce changes in the circulating levels of either GH, glucose, insulin, or IGF‐1.

In this study, we show that muscles of *Ercc1* ^*Δ/−*^ mice not only are smaller but contain numerous subcellular and biochemical abnormalities. It is well documented that contractile force normalized per mass is preserved when muscle undergoes regulated loss in weight.^60^ Here, we see that *Ercc1* ^*Δ/*−^ mice generated only 50% of normalized grip strength compared with controls but that this value was significantly improved by sActRIIB treatment. Abnormal specific force and relaxation times in the muscle of *Ercc1* ^*Δ/*−^ muscle, both features of structure alterations,^61^ were improved by sActRIIB treatment. We therefore suggest that sActRIIB not only prevents muscle from undergoing atrophy but also prevents the subcellular abnormalities. This is clearly evident in the TEM profiles of skeletal muscle, which show that every abnormal feature of *Ercc1* ^*Δ/−*^ muscle (sarcomeres and mitochondria) was largely prevented by the action of sActRIIB. We suggest that at least one key mechanistic driver in this process is the activation of the autophagic programme mediated by members of the FoxO family. Thus, we propose that abnormally low autophagic activity displayed in *Ercc1* ^*Δ/−*^ muscle leads to accumulation of p62 puncta and presumably abnormal mitochondria as well as through hyper‐activation of the UPR^MT^,^62^ elevated levels of genes encoding *prohibitins* that function to restore organelle function,^36^ change in the histone mark profile (down‐regulation of H3K9Me3 and up‐regulation of H4K20me3) as well as ROS superoxide levels. The build‐up of ROS causes protein oxidation ultimately compromising the workings of the contractile apparatus and leading to a deficit in specific force.^63^ Our interpretation of these finding is that sActRIIB treatment of *Ercc1* ^*Δ/−*^ mice leads to the activation of autophagy (LC3II/I ratio), which prevents the accumulation of p62 puncta and also abnormally functioning mitochondria, thus counteracting the need to activate either the UPR^MT^ or prohibitin programmes, maintains a normal profile of histone modification as well as avoiding the build‐up of high levels of ROS, which ultimately translates in the preservation of organ reserve capacity. We note that the expression of Mul1, a proposed mitochondrion‐specific U3 ubiquitin ligase, was not affected by the progeroid condition or following treatment with sActRIIB compared with controls. However, it is worth bearing in mind that numerous mitochondria targeting U3 ubiquitin ligases have been identified including PARKIN and that these non‐investigated molecules could be executing mitophagy in our experiments.^40^

However, the concomitant increase in protein synthesis and autophagy levels in *Ercc1* ^*Δ/−*^ mice following sActRIIB treatment remains to be further investigated. In a normal setting, the two processes are antagonistically driven in large part by FoxO proteins (reviewed in Bonaldo & Sandri^56^). Here, we suggest that normal parameters do not operate evidenced by the hyper‐activation of Akt in *Ercc1* ^*Δ/−*^ muscle. Indeed, there is a growing body of evidence for dual activation of Akt‐mediated pathways and autophagy when the normal landscape of regulation is altered.^64^ Hyper‐activation leading to initiation of novel signalling pathways and cellular outcomes is quite a common outcome and has been extensively studied especially in scenarios of uncontrolled cell division that underpin the development of many cancers.^65^, ^66^ Future studies, beyond the scope of the present investigation, combining gene expression and proteomic platforms are planned to identify the pathways susceptible to hyper‐activation of Akt. Nevertheless, we propose that the role of autophagy is in maintaining cellular homeostasis rather than anabolism.^26^

### Effects of soluble activin receptor type IIB treatment on muscle stem cells and the extra cellular matrix

Our work identifies novel features of progeroid SC, the resident stem cell population of skeletal muscle.^67^ We show that the number of SC is reduced by the *Ercc1* mutation and it renders the cells senescent gauged by their inability to proliferate following single fibre isolation. They were able to become activated, judged by their expression of MyoD (data not shown), and differentiate but did not demonstrate the normal self‐renewal/terminal differentiation physiognomies following 72 h of culture,^68^ features shared by counterparts from geriatric wild‐type mice.^35^ Our results are consistent with the findings of Lavasani *et al*. who showed that *Ercc1* ^*Δ/−*^ mice have attenuated muscle regeneration following cardiotoxin injury.^16^ The subsequent experiments reveal features of SC that are plastic with regard to myostatin/activin signalling. First, we show that sActRIIB was unable to influence the number of SC in the muscle of *Ercc1* ^*Δ/−*^ mutants, which remained abnormally low compared with controls. This is not altogether surprising as the number of SC is established approximately a month after birth in mice.^69^ However, sActRIIB treatment supported SC division and normal differentiation. We propose that these outcomes are unlikely to be due to a direct attenuation of myostatin/activin signalling in SC by sActRIIB as previous studies have shown that they express very little, if any, ActRIIB.^70^ Rather, we contemplate that sActRIIB‐induces change in the *Ercc1* ^*Δ/−*^ myofibre ECM (shown here by changes in collagen IV expression as well as dystrophin) that influences the behaviour of their SC. This is possibly significant given that recent studies have shown that the behaviour (ability to divide and differentiate) of SC is profoundly influenced by the interaction of collagen molecules and stem cell receptors.^71^

## Compression of morbidity by soluble activin receptor type IIB treatment

In this study, we show that antagonism of myostatin/activin signalling in *Ercc1* ^*Δ/−*^ mice attenuated the development of ageing‐related changes not only of muscle but rather also of health overall. Administration of sActRIIB to *Ercc1* ^*Δ/−*^ mice improved strength, fitness, and locomotor performance, delayed the onset and importantly the severity of several age‐related neurological abnormalities, and reduced deterioration of the bones, liver, and kidney.

An issue that needs addressing is how sActRIIB delivers multi‐organ protection against ageing. One possibility is that myostatin/activin signalling promotes the age‐related changes independently in each organ system examined in this study and that they are attenuated by systemic delivery of sActRIIB. This certainly could be the case for the kidney as we have seen that downstream activation of myostatin/activin pathways, identified through presence of pSmad2/3, in tissue from progeric mice were blocked by sActRIIB. Moreover, numerous studies have shown that activation of TGF‐ß signalling, which can result in the Smad2/3 phosphorylation, is able to induce ‘podocyte disease transformation’, an atypical form of epithelial mesenchymal transition.^72^ However, we were unable to detect significant levels of pSmad2/3 in the liver (concordant with the findings of others^73^), which nevertheless showed signs of being protected from the ageing process by sActRIIB. Therefore, we postulate that sActRIIB may act both directly (e.g. muscle and kidney) or indirect for other organ systems (e.g. the liver). For the indirect actions of sActRIIB, it is possible that the skeletal muscle plays a role promoting the maintenance and homeostasis of other tissues through inter‐organ signalling. It is well known that pathology of skeletal muscle leads to failure of other organs. For example, rhabdomyolysis induces acute kidney injury in part by the release of myoglobin.^74^ There are also examples where a malfunctioning organ leads to myopathy, which in turn exacerbates the primary lesion. An example has been elegantly assimilated into the ‘Muscle Hypothesis’ of Chronic Heart Failure.^75^ Herein, changes in skeletal muscle structure and function, mediated by a number of factors including tissue hypoxia and inflammation, lead to hyper‐responsiveness of the ergoreflex system.^76^ This leads to over‐activation of the sympathetic nervous system and consequently an increased load on the ventricles.^77^ We postulate that severe *Ercc1* ^Δ/−^ muscle wasting results in the release of myokines and/or intracellular molecules, which lead to pathological changes in other organs.^78^ Additionally, we speculate that the normalization of aged muscle following treatment with sActRIIB may also impact the ergoreflex system. By protecting the muscle against age‐related changes through the action of sActRIIB, we propose a diminution in the release of harmful factors and possibly by changes to afferent activity. Support for our notion for the release of secreted factors comes from an elegant study that demonstrated transplantation of healthy muscle stem cells into a progeroid model led to the secretion of factors, which acted on numerous organs.^16^ Nevertheless, it is possible that sActRIIB mediates actions that are independent of skeletal muscle function. Indeed, it was recently reported that the same treatment used in our work attenuated hepatic protein synthesis and splenomegaly in a rodent cachexia model that were independent of changes in muscle phenotype.^79^

We are nevertheless mindful that despite promising results in rodent models, translation of therapies based on myostatin/activin antagonists have been, to date, unsuccessful in delivering intended outcomes and others have been curtained due to safety concerns^80^, ^81^ and point to the need to develop a greater understanding of the biological processes controlled by this signalling axis. A possible means of alleviating some of the safety issues associated with myostatin/activin antagonists could be through decreasing their dose but at the same time harnessing the benefits of other agents that promote healthy ageing. One attractive proposition could be to use a combination of myostatin/activin antagonists and the deployment of the angiotensin 1–7 hexapeptide. The latter has been shown to block over active renin‐angiotensin signalling, which not only drives muscle dysfunction but also leads to muscle fibrosis.^82^, ^83^ A recent study showed that angiotensin 1–7 was able to restore age‐related muscle weakness in a rodent model.^84^ Both the sActRIIB and angiotensin 1–7 are attractive therapeutic molecules because they could be delivered using existing medical devices such as osmotic mini‐pumps.

In conclusion, this dataset highlights a novel mechanism that attenuates age‐related tissues changes. Previous findings support the notion that an organism slows ageing by remodelling its cellular activity from growth and proliferation to maintenance and repair.^2^, ^3^, ^85^ This can be achieved by attenuating IGF‐1 and GH activity, which controls the somatic growth axis^86^, ^87^ and by dietary restriction (DR).^88^ We have previously shown that DR delays ageing at the organismal level and extends lifespan and health span of *Ercc1* ^Δ/−^ mice.^23^ These studies advocate that promoting tissue growth in an ageing model might well be harmful to the organism. However, we show that a mechanism that promoted growth of skeletal muscle also promotes overall health span as evidenced by activity measurements and tissue structure and function. We believe that we can reconcile these apparent discrepancies by examining the defects that underpin the accelerated ageing process in progeroid mice. We have shown that DNA repair deficiency leads to damage that stalls transcription at least in post‐mitotic tissues, which cannot dilute DNA damage by replication or repair it by replication‐associated repair pathways. As a consequence, the stochastic nature of DNA damage leads to a preferential loss of long transcripts.^23^ Furthermore, DR is able to counteract the transcriptional block by reducing the DNA damage load.^23^ We propose that, at least in muscle, the transcriptional landscape is unaltered by sActRIIB but that enhanced protein synthesis promoted by sActRIIB can compensate at least in part for the deficit in long transcripts by increasing the number of polypeptides from each mRNA molecule. In this model, the levels of proteins encoded by long genes in *Ercc1* ^Δ/−^ would be increased by either reduced arrest of gene transcription due to diminished DNA damage induction (by mechanisms induced by DR) or increased rate of protein synthesis (by sActRIIB). Future studies again relying on gene expression and proteomic platforms are planned to test this novel hypothesis.

In summary, we believe that attenuating myostatin/activin signalling protects numerous organs including the kidney, bone, liver, and likely nervous system as well as skeletal muscle through a combination of direct and inter‐organ signalling processes. Although delaying many aspects of ageing, overall lifespan was not increased by administration of sActRIIB. This implies that a model where attenuation of myostatin/activin signalling does not affect the upper limits of lifespan but rather compresses morbidity, sustaining health until very old ages.^89^, ^90^

## Author contributions

K. P., J. H. J. H., W. P. V., and T. B. H. performed conceptualization. K. P, J. H. J. H., and W. P. V. carried out methodology. W. P. V., R. M. C. B., N. v. V., and S. B. performed validation. K. P., W. P. V., K. A., and S. O. carried out formal analysis. K. A., W. P. V., S. O., O. K., M. H., F. S., B. J., R. M., T. M., A. P., O. R., A. M., and H. C.‐H. performed investigation. K. P., J. H. J. H., and W. P. V. carried out writing. K. A., S. O., K. P., and W. P. V. performed visualization. K. P., J. H. J. H., and W. P. V. carried out supervision.

## Conflict of interest

The authors declare no competing interests.

## Ethical statement

The authors certify that they comply with the ethical guidelines for publishing in the Journal of Cachexia, Sarcopenia and Muscle: update 2017.^24^

## Supporting information


**Figure S1.** Muscle profiling of 16‐week old male *Ercc1* ^*Δ/−*^ mice. (A) Muscle weights and normalized muscle weights to tibia length. (B) EDL and soleus fibre number count. (C) Frequency of centrally located nuclei in the EDL and soleus at 16 weeks. (D‐G) Muscle fibre cross sectional area in EDL, soleus and the deep and superficial regions of the TA in relation of MHC isoform expression. (H‐J) MHC isoform profile of EDL, deep and superficial regions of the TA. (K) Oxidative fibre number enumeration through histological SDH activity staining of the EDL and soleus. (L) Satellite cell and progeny enumeration on fresh and cultured EDL for 72 h. (M) Quantification of proportion of stem cells (Pax7^+^/Myogenin^−^) and differentiated cells (Pax7^−^/Myogenin^+^) on EDL fibres after 72 h culture. *n* = 6 male mice from each cohort for data presented in (A‐L). Fibres collected from 3 mice from each cohort and minimum of 25 fibres examined for (M). Students t‐test, * < 0.05, ** < 0.01, ***p < 0.001.

**Figure S2.** (A‐B) Body and (C‐D) organ weights from male control, untreated and sActRIIB treated Ercc1^Δ/−^ mice at end of 15 weeks age. One‐way ANOVA followed by Bonferronis multiple comparison tests, * < 0.05, ** < 0.01, ***p < 0.001.

**Figure S3.** Smad2/3 signalling, oxidative fibre number, caspase 3 expression and centrally located nuclei number changes induced by sActRIIB treatment in *Ercc1* ^*Δ/−*^ soleus without impacting on DNA damage. (A) Immunohistology of pSmad2/3 expression (green) in soleus muscle (yellow arrows). (B) Immunohistology of γH2A.X expression (green) in soleus muscle (yellow arrows). (C) Immunohistology of Caspase 3 expression in EDL and soleus muscle (red arrows). (D) H and E staining for the identification of centrally located nuclei in EDL and soleus muscle (black arrows). Scale for H and E 40 μm. (E) SDH stain in soleus of the three cohorts. Scale for SDH 80 μm. *N* = 8 male mice from each cohort. One‐way ANOVA followed by Bonferronis multiple comparison tests, * < 0.05, ** < 0.01, ***p < 0.001.

**Figure S4.** Western blotting demonstrating that sActRIIB promotes protein synthesis and autophagy but blunts proteasome protein breakdown in *Ercc1* ^*Δ/−*^ muscle. Immunoblots and densitometry quantification of (A) pAkt, (B) p4EBPI on Thr37/46 and Ser65, (C) pS6, (D) pFoxO1, (E) pFoxO3a, (F) LC3II/I, and (G) p62. (H) Densitometry quantification of eIF2α. (I‐K) qPCR quantification of *Atrogin‐1, MuRF1*, *Mul1* expression. (L) Quantification of p62 puncta. (M) Immunohistology of p62 puncta in the EDL muscle (green arrows) (N) Densitometry quantification of total puromycin incorporation (protein synthesis rate). (O) Densitometry quantification protein ubiquitination. *n* = 5 for all western blots and *n* = 8 for rest. Non‐parametric Kruskal‐Wallis test followed by the Dunns multiple comparisons used for (A‐H and N‐O). One‐way ANOVA followed by Bonferronis multiple comparison test used for (I‐K). * < 0.05, ** < 0.01, ***p < 0.001.

**Figure S5.** (A) *Ercc1* ^*Δ/−*^ kidney showing autophagosome (arrow). (B) Evidence for indirect action of sActRIIB in liver. pSmad2/3 (green) in relation to smooth muscle actin (red) in the three cohorts. Note that pSmad2/3 was very sparse in the three cohorts and when present was located adjacent to smooth muscle (arrow).

**Figure S6.** (A) μCT was used to locate and visualize the increase in muscle and bone volume in *Ercc1* ^*Δ/−*^ mice following sActRIIB treatment. Sex specific characterization of (B) body weights, (C) onset of sever tremors and (D) survival in the Dutch cohort. *n* = 5 for all three cohorts.

**Movie S1**. Representative film of control, *Ercc1* ^*Δ/−*^ and sActRIIB treated *Ercc1* ^*Δ/*−^ mice at 15 weeks of age.
