# Supplementary material for: Compression of morbidity in a progeroid mouse model through the attenuation of myostatin/activin signalling
Source: J Cachexia Sarcopenia Muscle. 2019 Mar 27;10(3):662–86. doi: 10.1002/jcsm.12404 (PMC6596402; doi:10.1002/jcsm.12404)
Supplement: Supplementary file 1 — Figure S1. Muscle profiling of 16‐week old male Ercc1 Δ/− mice. (A) Muscle weights and normalized muscle weights to tibia length. (B) EDL and soleus fibre number count. (C) Frequency of centrally located nuclei in the EDL and soleus at 16 weeks. (D‐G) Muscle fibre cross sectional area in EDL, soleus and the deep and superficial regions of the TA in relation of MHC isoform expression. (H‐J) MHC isoform profile of EDL, deep and superficial regions of the TA. (K) Oxidative fibre number enumeration through histological SDH activity staining of the EDL and soleus. (L) Satellite cell and progeny enumeration on fresh and cultured EDL for 72 h. (M) Quantification of proportion of stem cells (Pax7+/Myogenin−) and differentiated cells (Pax7−/Myogenin+) on EDL fibres after 72 h culture. n = 6 male mice from each cohort for data presented in (A‐L). Fibres collected from 3 mice from each cohort and minimum of 25 fibres examined for (M). Students t‐test, * < 0.05, ** < 0.01, ***p < 0.001. [file JCSM-10-662-s001.pdf]

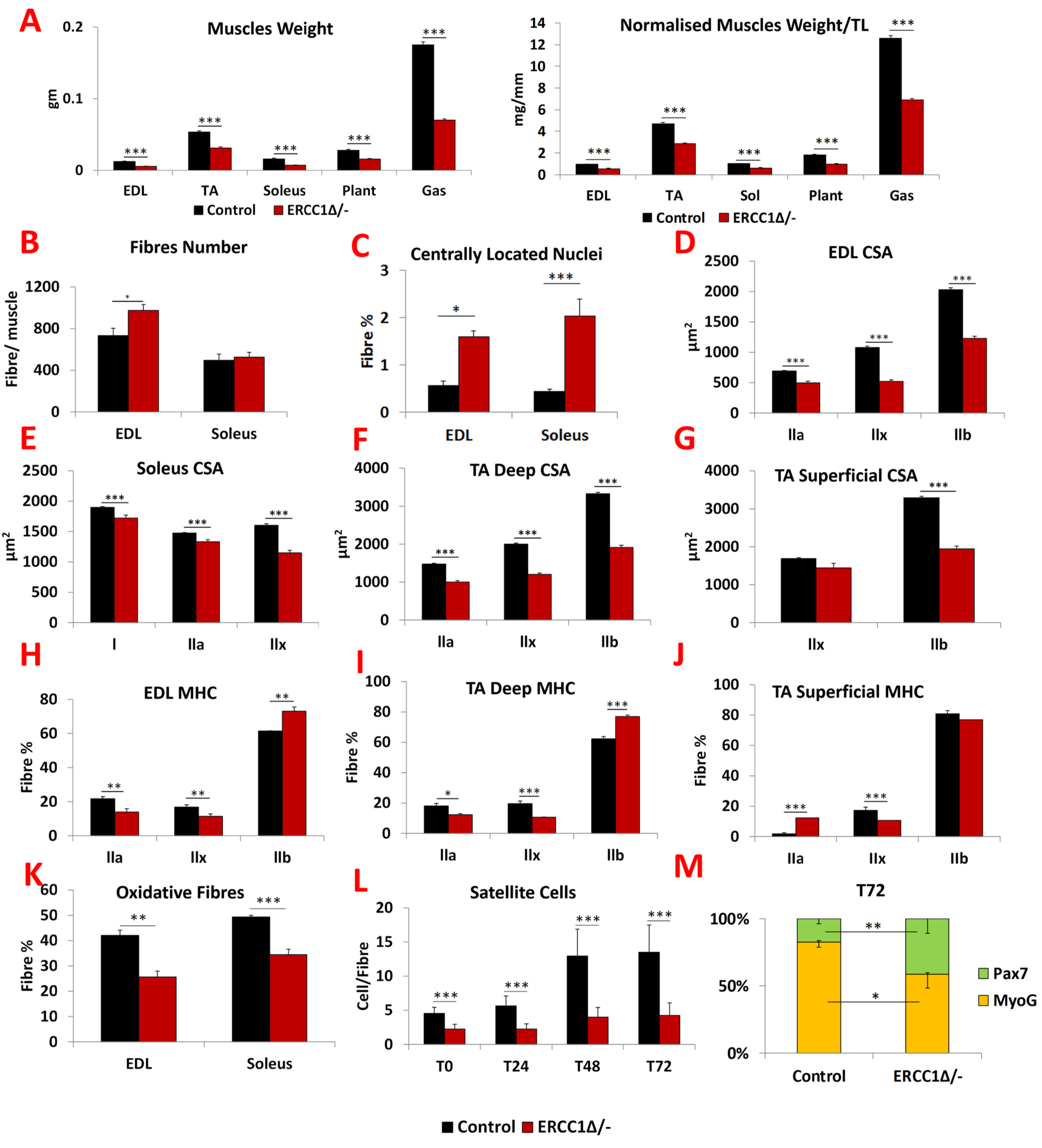

A
Muscles Weight
gm
0.2
0.1
0
EDL
TA
Soleus
Plant
Gas
Control
ERCC1Δ/-
Normalised Muscles Weight/TL
mg/mm
14
12
10
8
6
4
2
0
EDL
TA
Sol
Plant
Gas
Control
ERCC1Δ/-
B
Fibres Number
Fibre/ muscle
1200
800
400
0
EDL
Soleus
C
Centrally Located Nuclei
Fibre %
3
2
1
0
EDL
Soleus
D
EDL CSA
µm²
2500
2000
1500
1000
500
0
IIa
IIx
IIb
E
Soleus CSA
µm²
2500
2000
1500
1000
500
0
I
IIa
IIx
F
TA Deep CSA
µm²
4000
3000
2000
1000
0
IIa
IIx
IIb
G
TA Superficial CSA
µm²
4000
3000
2000
1000
0
IIx
IIb
H
EDL MHC
Fibre %
80
60
40
20
0
IIa
IIx
IIb
I
TA Deep MHC
Fibre %
100
80
60
40
20
0
IIa
IIx
IIb
J
TA Superficial MHC
Fibre %
100
80
60
40
20
0
IIa
IIx
IIb
K
Oxidative Fibres
Fibre %
60
50
40
30
20
10
0
EDL
Soleus
L
Satellite Cells
Cell/Fibre
20
15
10
5
0
T0
T24
T48
T72
M
T72
100%
50%
0%
Control
ERCC1Δ/-
Pax7
MyoG
Control
ERCC1Δ/-
