# Supplementary figures and images for: Compression of morbidity in a progeroid mouse model through the attenuation of myostatin/activin signalling
Source: J Cachexia Sarcopenia Muscle. 2019 Mar 27;10(3):662–86. doi: 10.1002/jcsm.12404 (PMC6596402; doi:10.1002/jcsm.12404)

**A** Change in Body weight Week 8 to 16

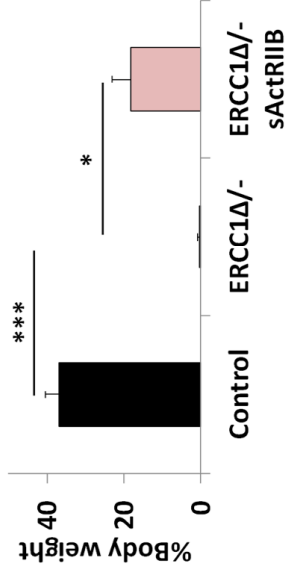

**B**

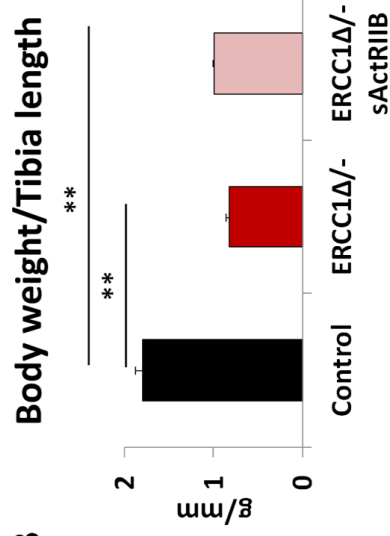

Organ weight

**C**

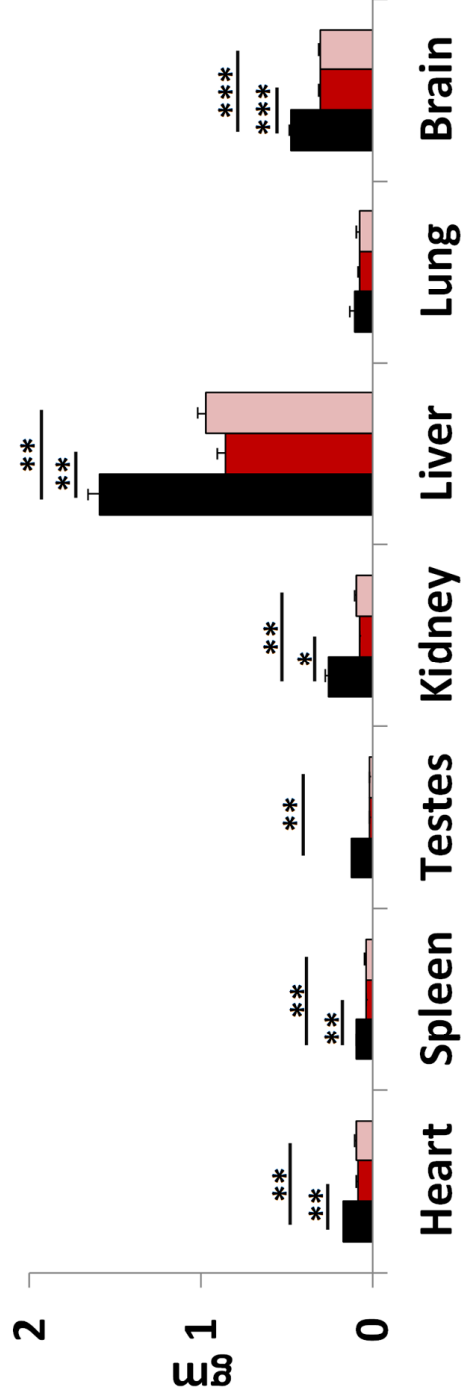

**D**

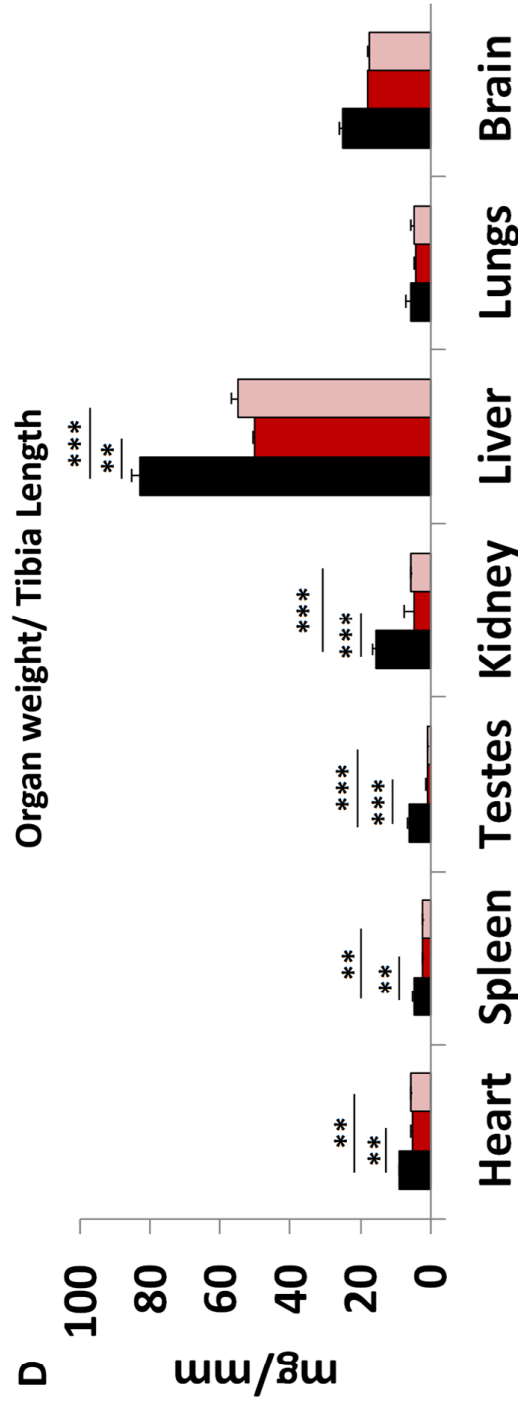

Supplement: Supplementary file 2 — Figure S2. (A‐B) Body and (C‐D) organ weights from male control, untreated and sActRIIB treated Ercc1Δ/− mice at end of 15 weeks age. One‐way ANOVA followed by Bonferronis multiple comparison tests, * < 0.05, ** < 0.01, ***p < 0.001. [file JCSM-10-662-s002.pdf]

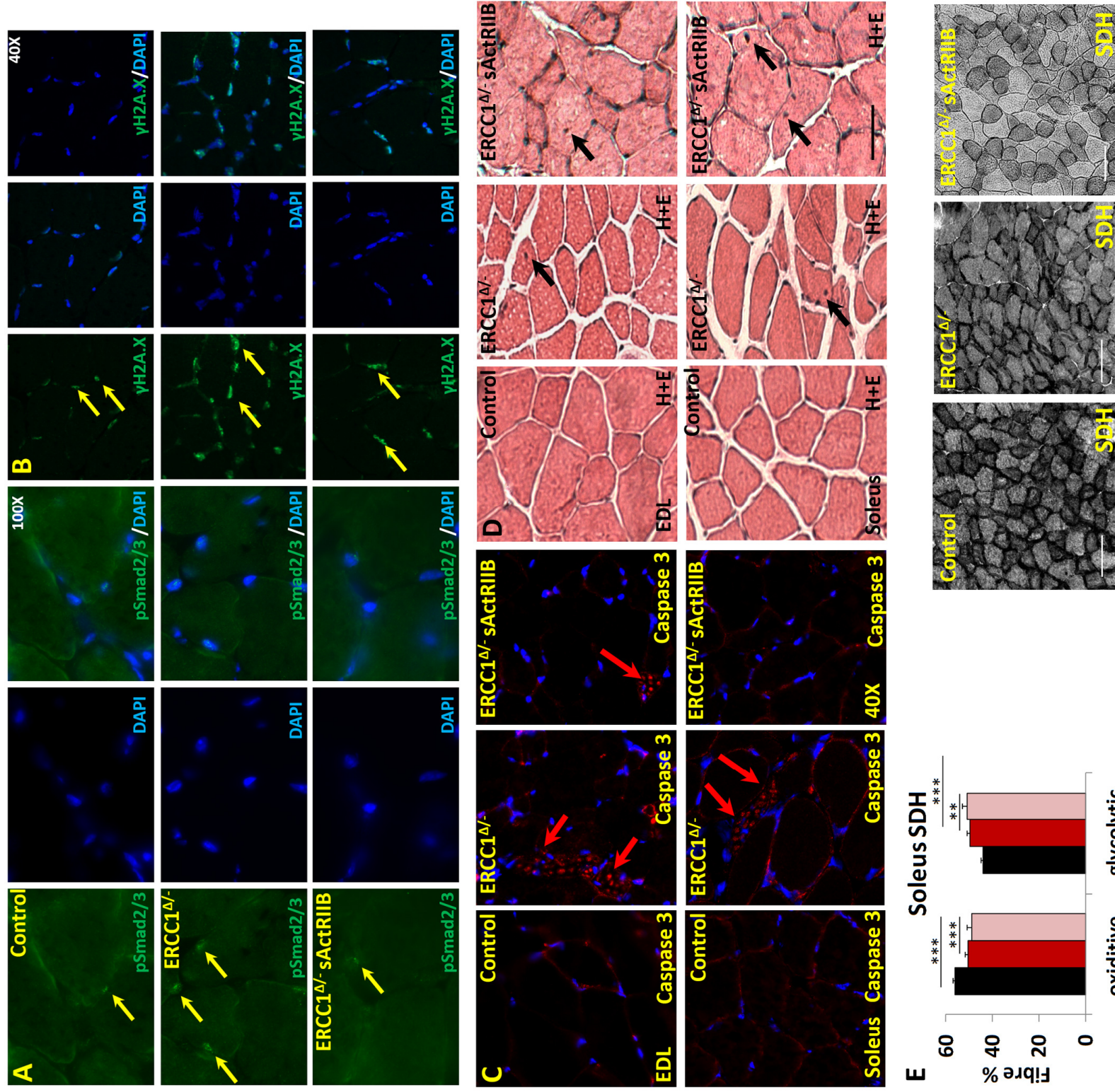

Supplement: Supplementary file 3 — Figure S3. Smad2/3 signalling, oxidative fibre number, caspase 3 expression and centrally located nuclei number changes induced by sActRIIB treatment in Ercc1 Δ/− soleus without impacting on DNA damage. (A) Immunohistology of pSmad2/3 expression (green) in soleus muscle (yellow arrows). (B) Immunohistology of γH2A.X expression (green) in soleus muscle (yellow arrows). (C) Immunohistology of Caspase 3 expression in EDL and soleus muscle (red arrows). (D) H and E staining for the identification of centrally located nuclei in EDL and soleus muscle (black arrows). Scale for H and E 40 μm. (E) SDH stain in soleus of the three cohorts. Scale for SDH 80 μm. N = 8 male mice from each cohort. One‐way ANOVA followed by Bonferronis multiple comparison tests, * < 0.05, ** < 0.01, ***p < 0.001. [file JCSM-10-662-s003.pdf]

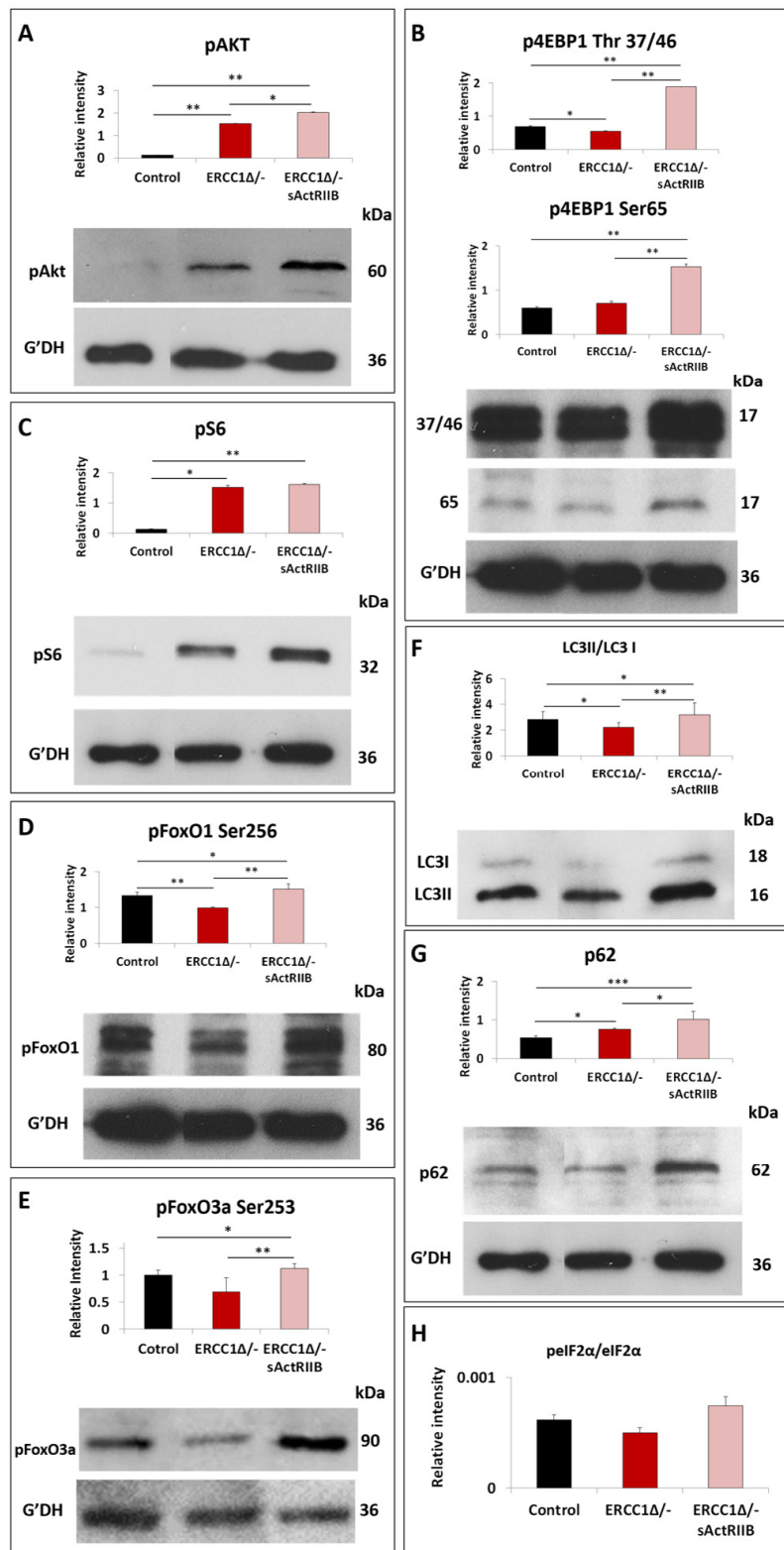

Supplement: Supplementary file 4 — Figure S4. Western blotting demonstrating that sActRIIB promotes protein synthesis and autophagy but blunts proteasome protein breakdown in Ercc1 Δ/− muscle. Immunoblots and densitometry quantification of (A) pAkt, (B) p4EBPI on Thr37/46 and Ser65, (C) pS6, (D) pFoxO1, (E) pFoxO3a, (F) LC3II/I, and (G) p62. (H) Densitometry quantification of eIF2α. (I‐K) qPCR quantification of Atrogin‐1, MuRF1, Mul1 expression. (L) Quantification of p62 puncta. (M) Immunohistology of p62 puncta in the EDL muscle (green arrows) (N) Densitometry quantification of total puromycin incorporation (protein synthesis rate). (O) Densitometry quantification protein ubiquitination. n = 5 for all western blots and n = 8 for rest. Non‐parametric Kruskal‐Wallis test followed by the Dunns multiple comparisons used for (A‐H and N‐O). One‐way ANOVA followed by Bonferronis multiple comparison test used for (I‐K). * < 0.05, ** < 0.01, ***p < 0.001. [file JCSM-10-662-s004.pdf]

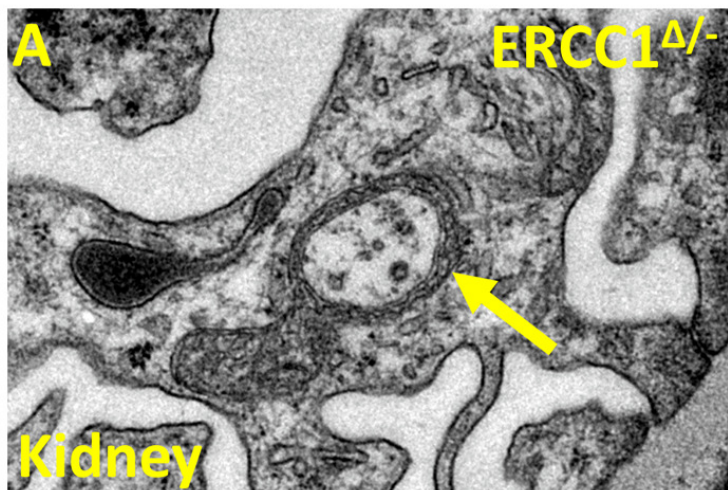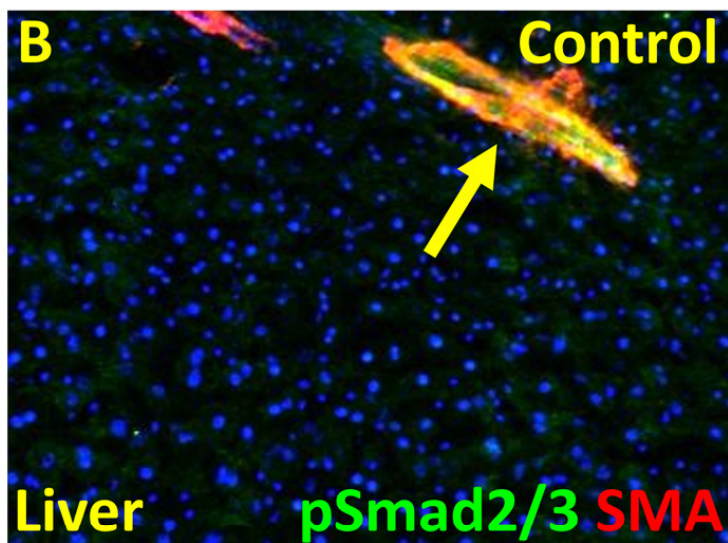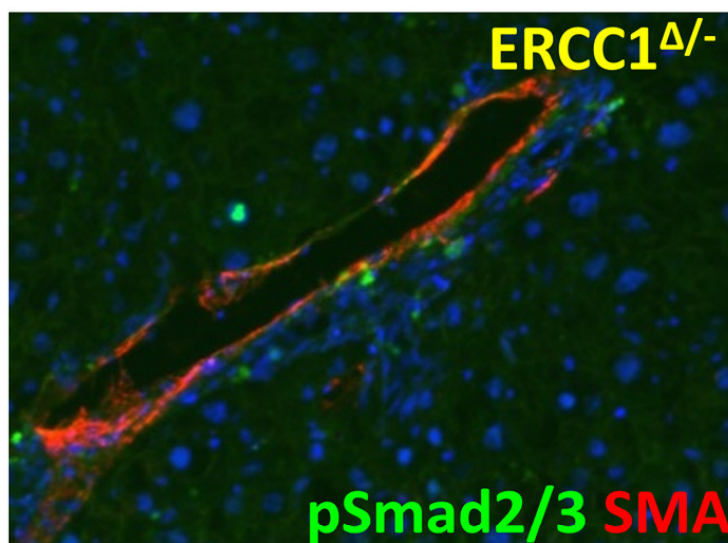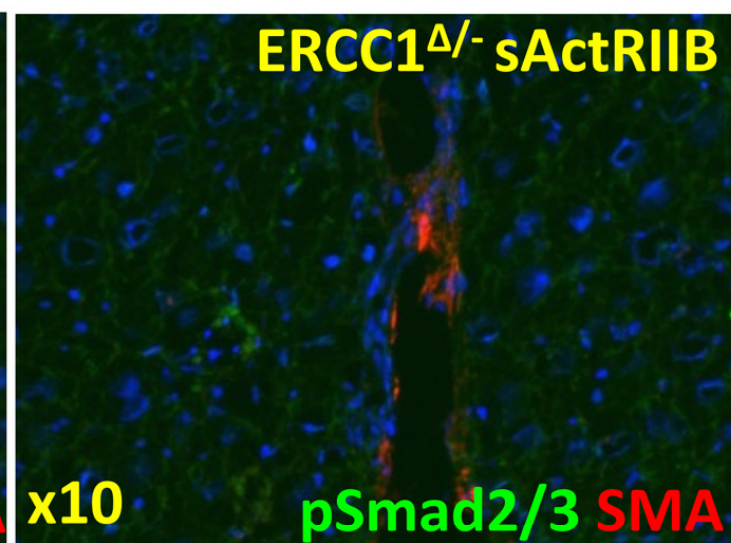

Supplement: Supplementary file 5 — Figure S5. (A) Ercc1 Δ/− kidney showing autophagosome (arrow). (B) Evidence for indirect action of sActRIIB in liver. pSmad2/3 (green) in relation to smooth muscle actin (red) in the three cohorts. Note that pSmad2/3 was very sparse in the three cohorts and when present was located adjacent to smooth muscle (arrow). [file JCSM-10-662-s005.pdf]
