# Supplementary material for: Compression of morbidity in a progeroid mouse model through the attenuation of myostatin/activin signalling
Source: J Cachexia Sarcopenia Muscle. 2019 Mar 27;10(3):662–86. doi: 10.1002/jcsm.12404 (PMC6596402; doi:10.1002/jcsm.12404)
Supplement: Supplementary file 6 — Figure S6. (A) μCT was used to locate and visualize the increase in muscle and bone volume in Ercc1 Δ/− mice following sActRIIB treatment. Sex specific characterization of (B) body weights, (C) onset of sever tremors and (D) survival in the Dutch cohort. n = 5 for all three cohorts. [file JCSM-10-662-s006.pdf]

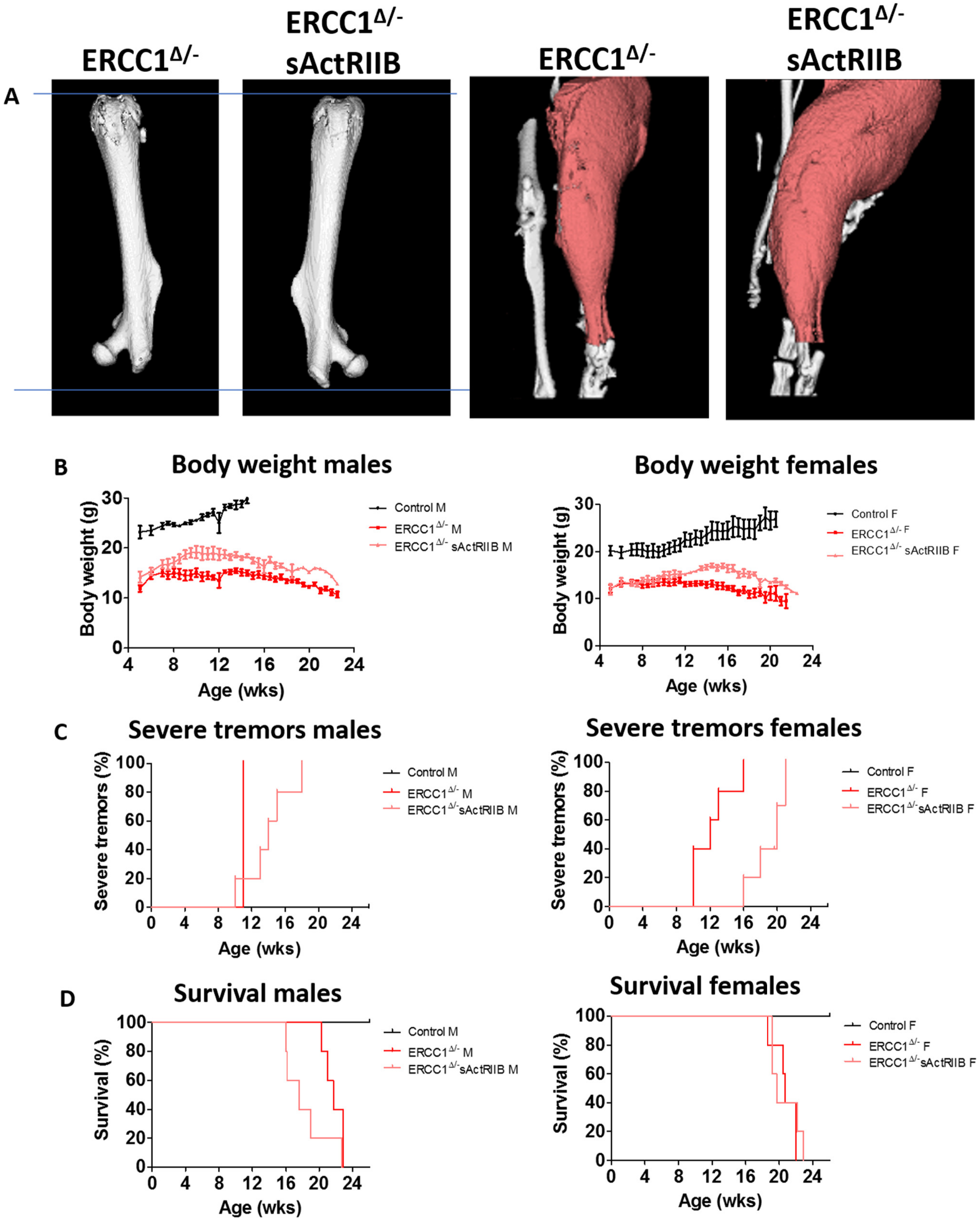
A
ERCC1$^{\Delta/-}$
ERCC1$^{\Delta/-}$ sActRIIB
ERCC1$^{\Delta/-}$
ERCC1$^{\Delta/-}$ sActRIIB
B
Body weight males
Body weight (g)
Age (wks)
Control M
ERCC1$^{\Delta/-}$ M
ERCC1$^{\Delta/-}$ sActRIIB M
Body weight females
Body weight (g)
Age (wks)
Control F
ERCC1$^{\Delta/-}$ F
ERCC1$^{\Delta/-}$ sActRIIB F
C
Severe tremors males
Severe tremors (%)
Age (wks)
Control M
ERCC1$^{\Delta/-}$ M
ERCC1$^{\Delta/-}$sActRIIB M
Severe tremors females
Severe tremors (%)
Age (wks)
Control F
ERCC1$^{\Delta/-}$ F
ERCC1$^{\Delta/-}$sActRIIB F
D
Survival males
Survival (%)
Age (wks)
Control M
ERCC1$^{\Delta/-}$ M
ERCC1$^{\Delta/-}$sActRIIB M
Survival females
Survival (%)
Age (wks)
Control F
ERCC1$^{\Delta/-}$ F
ERCC1$^{\Delta/-}$sActRIIB F
